# Molybdenum disulfide induces growth inhibition and autophagy-dependent hepatocyte cell death through directly binding and regulating the activity of MST2

**DOI:** 10.1016/j.mtbio.2025.102394

**Published:** 2025-10-08

**Authors:** Zijuan Qi, Yuanliang Yan, Zhijie Xu, Wei Chong, Yuchen Qiu, Xiaofeng Huang, Jiajun Jing, Huancai Fan, Qiuju Liang, Sijin Liu, Li Yan, Leping Li, Ming Gao

**Affiliations:** a Department of Gastrointestinal Surgery, Shandong Provincial Hospital Affiliated to Shandong First Medical University, Jinan, 250021, Shandong, China; b State Key Laboratory of Environmental Chemistry and Ecotoxicology, Research Center for Eco-Environmental Sciences, Chinese Academy of Sciences, Beijing, 100085, China; c University of Chinese Academy of Sciences, Beijing, 100049, China; d Department of Pharmacy, Xiangya Hospital, Central South University, Changsha, 410008, Hunan, China; e Department of Pathology, Xiangya Hospital, Central South University, Changsha, 410008, Hunan, China; f College of Environmental and Resource Sciences, Fujian Normal University, Fuzhou, 350117, Fujian, China

**Keywords:** MoS_2_ nanosheets, Hepatotoxicity, CRISPR-Cas9, MST2 protein

## Abstract

Two-dimensional sheet-like nanomaterial molybdenum disulfide (MoS_2_) has extensive potential applications in the biomedical field. Nevertheless, upon entering the body, MoS_2_ tends to accumulate markedly in the liver, drawing increasing attention to its potential hepatotoxicity. In this study, we demonstrated that MoS_2_ nanosheets exerted cytotoxic effects on the liver both *in vitro* and *in vivo*. Further exploration of the toxicity mechanism, utilizing genome-wide CRISPR-Cas9 screening and molecular biological techniques, uncovered that MST2 protein is crucial in mediating MoS_2_-induced cytotoxicity. Moreover, protein mass spectrometry and molecular dynamics simulation results indicated that MoS_2_ directly binds to MST2 protein, thereby promoting its phosphorylation and activation. Subsequently, phosphorylated MST2 protein activates the Hippo signaling pathway, which in turn suppresses liver cell proliferation. Our *in vivo* experiments revealed that MST2 is indispensable for MoS_2_-induced impairment of liver regeneration in mice and disordered liver development in zebrafish. Meanwhile, the MoS_2_/MST2 axis was to found induce hepatocyte death *via* activation of autophagy dependent on LC3 protein. Collectively, this study provides a foundation for a comprehensive understanding of the biological behavior and hepatotoxicity of MoS_2_, offering valuable insights for the safety assessment of nanomaterials and the rational design of future nanomedicines.

## Highlights

• MoS_2_ exposure induces significant hepatotoxicity in both *in vitro* and *in vivo* models.
• MoS_2_ inhibits cell proliferation through MST2-mediated mechanisms.
• MoS_2_ induces autophagic cell death *via* MST2 signaling pathways.
• MST2 is essential for MoS_2_ mediating acute liver injury and impaired liver regeneration.

## Graphical abstract

**Figure:**
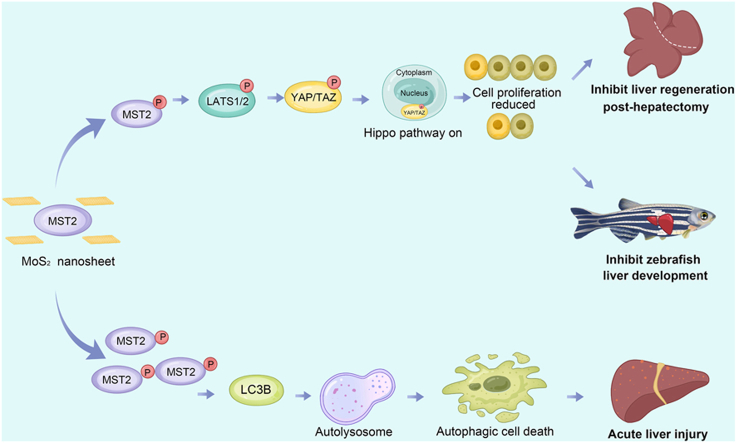

## 1 Introduction

Molybdenum disulfide (MoS_2_) nanosheets is a class of typical transition metal dichalcogenide, characterized by an S-Mo-S layered structure where molybdenum bonded on either side to layers of sulfur [1]. MoS_2_ nanosheets have the potential for widespread application in biomedical fields such as imaging, diagnosis, drug delivery, and photothermal therapy due to their unique physicochemical/biological properties. For example, because of the high photothermal conversion efficiency *in vitro*, MoS_2_ significantly improves photothermal therapy effect and can act as the photothermal agent; the large surface-to-volume ratio allows MoS_2_ to provide numerous binding sites for loading drug molecules, which combines chemotherapy and photothermal therapy to produce efficient synergistic therapeutic outcomes; MoS_2_ nanosheets can be also used as sensors for detecting biomolecules such as DNA or enzyme *in vivo* due to its fluorescence-quenching ability [2].

Compatibility with biological systems is the first critical determinant for the clinical application of the biocomposite materials based on MoS_2_ nanosheets. Although the tissue biodistribution, cellular uptake, intracellular localization, transformation, and degradation of MoS_2_ have been studied in previous reports, the cytotoxic effect and related mechanism of MoS_2_ were still obscure [[3], [4], [5]]. It was reported that after intravenous injection of mice with MoS_2_, soluble molybdenum was primarily accumulated in the liver compared to other tissues, which caused potential damage to the liver [3]. However, the understanding of the hepatotoxicity and related mechanism of MoS_2_ are remains limited. It was reported that MoS_2_ significantly inhibited the activity of transmembrane ATP binding cassette efflux transporter in liver cells at low concentrations, while high concentrations of MoS_2_ damaged mitochondrial membrane potential and integrity, which result in cytotoxicity [6]; functionalized MoS_2_ nanosheets at a concentration of 20 mg/L were observed to elicit a robust inflammatory response and cause notable focal damage in zebrafish liver tissues, characterized by peripheral nuclei and vacuolation [7]. However, absence of the knowledge regarding the biotarget of MoS_2_ in the liver poses a significant challenge for deeply evaluating the biosafety of MoS_2_ in clinical application.

Nanosheets has large surface area and high surface free energy, which enable internalized nanosheets rapidly adsorb proteins onto their surfaces. Hydrophobic interactions primarily initiate the process of protein anchoring to the nanosheet surface, and van der Waals forces and electrostatic interactions stabilize the nanosheet-protein binding. The interaction between nanosheet and adsorbed proteins can regulate the conformation and biological functions of proteins, thereby determining the health risk of nanosheets. For instance, upon interacting with MoS_2_, the tertiary structure and the local secondary structure of human serum albumin, transferrin, fibrinogen and globular protein HP35 were changed, which subsequently affected downstream cellular signaling processes [8,9]. Fullerene C60 nanocrystals could increase the autonomous activity and the level of autophosphorylation of Ca (2+)/calmodulin-dependent protein kinase II, a multimeric intracellular serine/threonine kinase, and then triggered time-dependent activation of ERK and CREB [10]. Therefore, an in-depth analysis of MoS_2_-binding functional proteins is the critical step for understanding the essence of biological fate of effects mediated by MoS_2_.

MST2 (STK3) is a serine/threonine kinase that plays a pivotal role in the Hippo signaling pathway, regulating gene transcription involved in cell growth, survival, apoptosis, proliferation, and organ size control. Autophosphorylation of MST2 is a key event in the Hippo signaling cascade, which subsequently triggers the activation of the NDR kinase family members LATS1/2 and modulates the YAP/TAZ complex. When the Hippo pathway is inactive, YAP moves to the cell nucleus and associates with the TEAD family of transcriptional regulators, resulting in the activation of genes that are crucial for cellular processes such as survival, expansion, and multiplication. In our study, we investigated the cytotoxic effects and aspects of MoS_2_ on the liver, and identified that MST2 is responsible for the toxic effects of MoS_2_ and could directly interact with MoS_2_. Further mechanism exploration showed that MoS_2_ inhibited liver cell proliferation, mice liver regeneration and zebrafish liver development by regulating MST2-YAP pathway; meanwhile, MoS_2_ induced the increase of LC3B-II levels, autophagy-dependent cell death and mice acute liver injury through activating MST2. Together, this study provides an overall understanding of the molecular mechanisms underlying the hepatotoxicity of MoS_2_, and suggests that design the MoS_2_-based nanocomposite that avoiding activating MST2 is a promising strategy to ensure the safer clinical application of MoS_2_ in the future.

## 2 Results

### 2.1 Characterization of MoS_2_

**Fig. 1:**
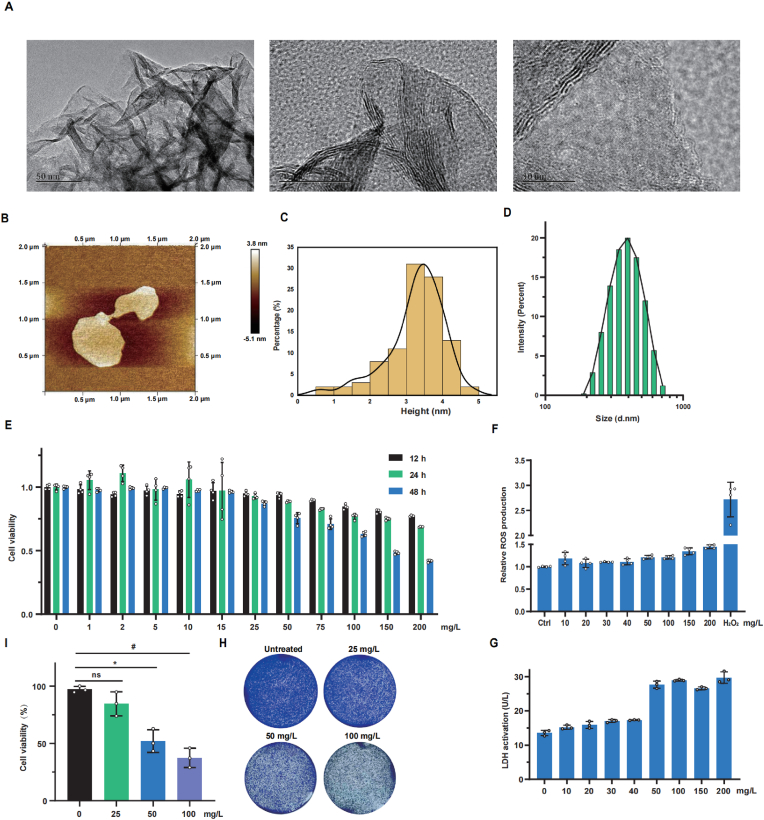
**Characterization and cytotoxicity of MoS**_**2**_**nanosheets.** (A) TEM images of the structure of MoS_2_ nanosheets at different magnification (scale bar = 50 nm, scale bar = 20 nm, scale bar = 10 nm). (B) AFM images of the structure of MoS_2_ nanosheets. (C) Histogram of the height of the MoS_2_ nanosheets according to the quantification of AFM images (n = 100). (D) The distributions of the hydrodynamic diameters of the MoS_2_ nanosheets in water. (E) Cell viability of HepG2 cells after treatment with the indicated concentration of MoS_2_ nanosheets for 12, 24, 48 h were measured by CCK-8 assay (n = 4). (F) Relative ROS production of HepG2 cells after treatment with the indicated dosage of the MoS_2_ nanosheets was determined by using H_2_DCFDA (n = 4). (G) The membrane integrity of HepG2 cells after 24 h of treatment with the indicated dosage of the MoS_2_ nanosheets was measured by LDH Assay Kit (n = 3). (H) Crystal violet staining of the HepG2 cells treatment with the indicated dosage of the MoS_2_ nanosheets. (I) Trypan blue exclusion of the HepG2 cells treatment with the indicated dosage of the MoS_2_ nanosheets (n = 3). A two-sided Student's t-test was used to determine p-values (∗p < 0.05, #p < 0.01). (For interpretation of the references to colour in this figure legend, the reader is referred to the Web version of this article.)

TEM and AFM analyses were conducted to examine the morphology of synthesized MoS_2_ nanosheets. The TEM images revealed that the MoS_2_ nanosheets were free-standing with a sheet-like structure (Fig. 1A). AFM measurements, including height profiling, indicated that the thickness of the MoS_2_ nanosheets was less than 5 nm (Fig. 1B and C). Furthermore, the size distribution and zeta-potential of nanosheets was assessed using dynamic light scattering (DLS) (Fig. 1D), yielding an average hydrated particle size of 398.33 ± 26.73 nm. And the zeta-potential of MoS_2_ nanosheets in water is −23.5 ± 0.5 mV.

### 2.2 MoS_2_ induces hepatotoxicity

To evaluate the potential adverse effects of MoS_2_ nanosheets on liver cells, HepG2 cells were treated with varying concentrations of MoS_2_ for 12, 24, and 48 h. As shown in Fig. 1E, cell viability was significantly reduced after exposure to MoS_2_ nanosheets at concentrations above 50 mg/L for 24 and 48 h. Meanwhile, reactive oxygen species (ROS) production was only slightly increased in response to MoS_2_ (Fig. 1F). In addition, MoS_2_ nanosheets also induced an increase of lactate dehydrogenase (LDH) release in the medium (Fig. 1G), indicating more cell membrane damage with increasing MoS_2_ concentrations. Moreover, the results of crystal violet staining and trypan blue exclusion indicated that the cell survival percentage were significantly gradually reduced accompanied with the increased concentration of MoS_2_ nanosheets (Fig. 1H and I). Together, these results suggested that MoS_2_ could cause hepatotoxicity in a ROS independent manner.

### 2.3 The internalization of MoS_2_ nanosheets leads to cytotoxicity

**Fig. 2:**
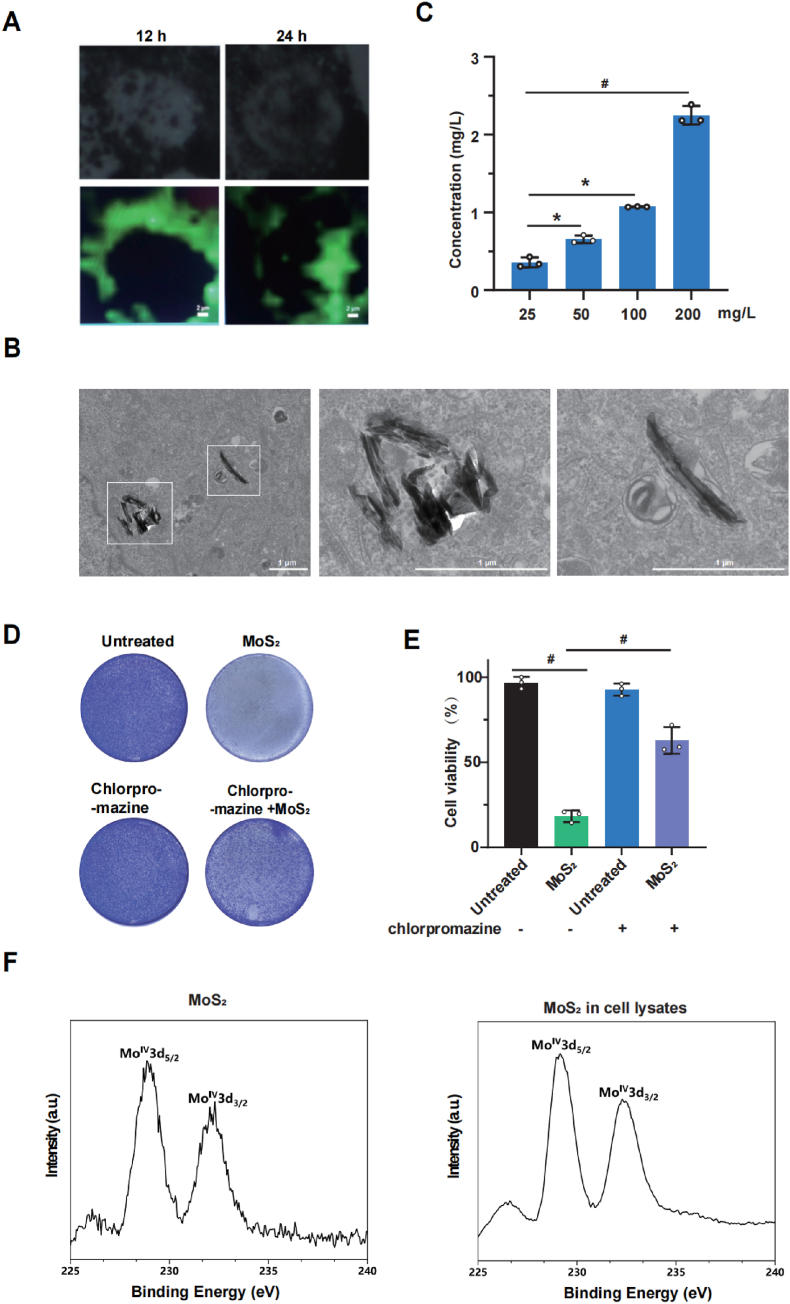
**The internalization of MoS**_**2**_**nanosheets into cells leads to cytotoxicity.** (A) Cellular Raman imaging of the HepG2 cells after treatment with the MoS_2_ nanosheets for 12 h and 24 h by using a confocal Raman spectrometer, the green fluorescence indicates the MoS_2_. Scale bar = 2 μm. (B) The imaging of the MoS_2_ nanosheets entering HepG2 cells was obtained by an Electron Microscopy. Scale bar = 1 μm. (C) The Mo^4+^ concentration in the lysates of cells exposed to MoS_2_ solutions at various concentrations for 24 h were measured by ICP-MS (n = 3). (D) Crystal violet staining of the HepG2 cells treatment with the MoS_2_ nanosheets and clathrin-mediated endocytosis inhibitor. (E) Trypan blue exclusion of the HepG2 cells treatment with the MoS_2_ nanosheets and clathrin-mediated endocytosis inhibitor (n = 3). (F) XPS spectra of pristine and internalized MoS_2_ nanosheets (the analyzer mode was CAE, the X-ray source was Al K Alpha). A two-sided Student's t-test was used to determine p-values (∗p < 0.05, #p < 0.01). (For interpretation of the references to colour in this figure legend, the reader is referred to the Web version of this article.)

To investigate whether MoS_2_-induced cytotoxicity was attributed to its cellular internalization, cellular Raman imaging was performed after MoS_2_ exposure for 12 and 24 h. As shown in Fig. 2A, MoS_2_ having strong green fluorescence was obvious internalized into cells. In addition, electron microscope pictures and ICP-MS data showed that there have internalized MoS_2_ nanosheets in cells (Fig. 2B–C). Furthermore, a series of endocytosis inhibitors was utilized to block the cellular internalization process, including chlorpromazine and Dynasore (both serving as clathrin-mediated endocytosis inhibitors), Genistein (a caveolin-mediated endocytosis inhibitor), methyl-β-cyclodextrin (MβCD, a lipid raft-mediated endocytosis inhibitor), Cytochalasin D (an inhibitor of F-actin polymerization), and EIPA (a macropinocytosis inhibitor). As shown in Fig. 2D–E and Fig. S1A–J, chlorpromazine and Dynasore, rather than the inhibitors targeting other endocytosis pathways, significantly mitigated MoS_2_-induced cell death. This observation indicates that clathrin-mediated endocytosis is essential for the subsequent cytotoxicity induced by MoS_2_ nanosheets. Previous studies have shown that MoS_2_ may be oxidized in the liver where molybdenum is chemically transformed from Mo (IV) to Mo (VI) and the intermediate chemical form Mo (V) [3]. Thus, this study utilized XPS to determine the oxidation state of MoS_2_ in liver cells. As shown in Fig. 2F, there was no significant alteration in the oxidation state of molybdenum in internalized MoS_2_ compared to that in pristine MoS_2_, which confirms that the cytotoxicity of MoS_2_ is not induced by the alteration of its oxidation state. To further confirm whether MoS_2_-induced hepatotoxicity was due to its release of molybdenum ion, we assessed the dissolved molybdenum ion content in PBS and DMEM culture media following the addition of 200 mg/L MoS_2_ for 24 h. The results (Fig. S1K) indicated that molybdenum ion content in the culture media was approximately 300 μg/L. However, even 10 mg/L molybdenum ions in medium did not cause significant cell death, indicating that the cytotoxicity of MoS_2_ is not attributed to the dissolved molybdenum ions (Fig. S1L).

### 2.4 Genome-wide CRISPR screen identifies MST2 as a crucial target for MoS_2_-induced cytotoxicity

**Fig. 3:**
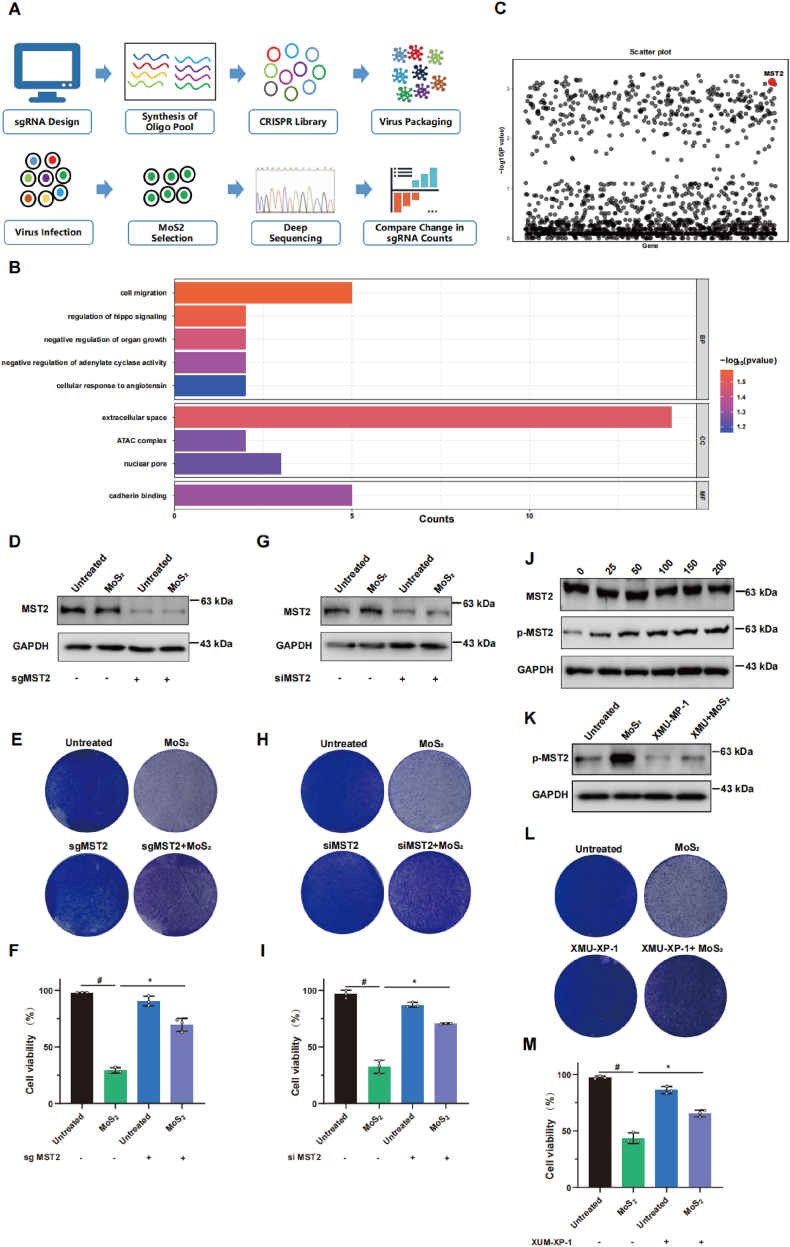
**Genome-wide CRISPR screening identifies MST2 as a crucial gene for MoS**_**2**_**-induced cytotoxicity.** (A) Flowchart of genome-wide CRISPR screening of MoS_2_-induced cytotoxicity -associated genes using the pooled human GeCKOv2.0 lentivirus sgRNA library. (B) GO analysis of the top 100 negatively selected hits in the CRISPR-Cas9 screen. (C) Scatterplot depicting results for MoS_2_ negatively selected hits in the CRISPR-Cas9 screen. (D–I) Control or MST2-depleted cells by sgRNA (D–F), siRNA (G–I) were treated with 100 mg/L MoS_2_ nanosheets, the protein level of MST2 was then measured by Western blotting assay, the immunoblotting experiments were performed 3 times independently(D, G), and the living cells were observed by crystal violet staining and trypan blue exclusion (E, F, H, I). (J) The protein levels of MST2 and phosphorylation MST2 in control and cells that were exposed to indicated concentration of MoS_2_ nanosheets were measured by Western blotting assay, and the experiments were performed 3 times independently. (K–M) The cells of control or inhibited phosphorylation MST2 (XMU-XP-1 pretreated) were treated with 100 mg/L MoS_2_ nanosheets, (K) the protein level of phosphorylation MST2 was then measured by Western blotting, the experiments were performed 3 times independently. (L–M) The living cells were observed by crystal violet staining and trypan blue exclusion. A two-sided Student's t-test was used to determine p-values (∗p < 0.05, #p < 0.01). (For interpretation of the references to colour in this figure legend, the reader is referred to the Web version of this article.)

To identify the key genes involved in MoS_2_-induced cytotoxicity, we performed a genome-wide CRISPR screen by using an sgRNA lentiviral library comprising 123,411 unique sgRNAs targeting 20,914 human genes (Fig. 3A). To systematically understand the molecular events associated with cytotoxicity, Gene Ontology (GO) analysis was performed on the top 100 enrichment genes (Fig. 3B). GO analysis showed that multiple biological processes participated in hepatotoxicity including regulation of Hippo signaling and negative regulation of developmental growth. These results were consistent to previous observations that MoS_2_ response was intimately linked to cell proliferation. Negative screen analysis was both performed based on MAGeCK algorithm, MST2 gene in Hippo pathway was identified as prominent candidate due to it was highly ranked in remains sgRNAs (Fig. 3C). Because MST2 is integral to Hippo signaling pathway and regulates cellular processes including proliferation, apoptosis, and tissue homeostasis, we thus selected MST2 for further investigation to ascertain its role as a potential target in MoS_2_ nanosheet-induced cytotoxicity. As shown in Fig. 3D–I, when MST2 were succeed knocked out by its sgRNA or knocked down by its siRNAs, MoS_2_-induced cells death was also attenuated. In addition, the protein level of MST2 after exposure to MoS_2_ nanosheets was not changed, whereas the phosphorylation level was markedly increased, suggesting that MoS_2_ influences the phosphorylation and activation of the MST2 (Fig. 3J). To test this hypothesis, MST2 inhibitor XMU-XP-1 was used to inhibit the phosphorylation of MST2. As shown in Fig. 3K–M, pretreatment with XMU-MP-1 prior to MoS_2_ exposure led to a significant decrease of MST2 phosphorylation and cell death percentage.

### 2.5 MoS_2_ nanosheets bind to MST2 protein and increase its phosphorylation

**Fig. 4:**
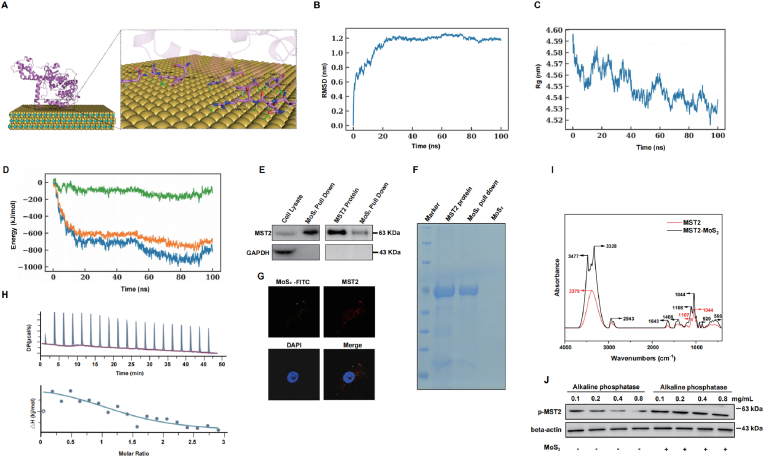
**MoS**_**2**_**nanosheets bind to MST2 protein and increase its phosphorylation.** (A) Molecular dynamics (MD) simulation model illustrating MoS_2_ nanosheets binding to MST2. (B–C) Profiles of root-mean-square deviation (RMSD; B), radius of gyration (Rg; C), and interaction energy (D) for MST2-MoS_2_ during a 100-ns MD simulation. Coul-SR: Coulomb Short-Range; Lennard-Jones Short-Range: LJ-SR. (E–F) Cell lysate and purified MST2 protein were separately incubated with 100 mg/L MoS_2_ nanosheets; thereafter the hard-corona complexes were isolated by washing and membrane ultrafiltration, the protein that bind to MoS_2_ nanosheets was detected by Western blotting assay (E) and Coomassie blue Staining (F), the experiments were performed 3 times independently. (G) HepG2 cells were added with 100 mg/L FITC-MoS_2_, then the representative images of MST2 (red) and FITC-MoS_2_ (green) were obtained by immunofluorescence analysis. Scale bar = 10 μm. (H) The result of interactions of MoS_2_ nanosheets and MST2 protein were determined by ITC. Real-time ITC thermogram of interactions of MoS_2_ nanosheets and MST2 proteins integrated heat results. The concentration of MoS_2_ nanosheets in the syringe is equal to 100 mM, and the concentrations of proteins in the cell are equal to 20 μM in the titrations. (I) MoS_2_ nanosheets (1 mg/mL) and MST2 protein (2 mg/mL) were incubated at 37 °C for 1 h. The resulting MoS_2_–protein complexes were isolated through washing and membrane ultrafiltration, followed by overnight drying and subsequent characterization by FTIR. (J) HepG2 cells that overexpressing MST2 proteins were treated with PBS or 100 mg/L MoS_2_ nanosheets for 24 h, and then the cell lysate was co incubated with alkaline phosphatase. The protein levels of the phosphorylation MST2 were detected by Western blot analysis, the experiments were performed 3 times independently. (For interpretation of the references to colour in this figure legend, the reader is referred to the Web version of this article.)

To explore how MoS_2_ nanosheets affects the phosphorylation of MST2 protein, we performed protein mass spectrometry assay to detect whether MoS_2_-absorbed proteins contribute to the activation of MST2. As shown in Fig. S2A, MoS_2_ nanosheets could adsorb many proteins on the surface analyzed by the Coomassie blue staining; moreover, the results of Tandem mass spectrum showed that the peptide of MST2 protein was also enriched among MoS_2_-pulldowned proteins (Fig. S2B and Table S1). To characterize the MST2–MoS_2_ interaction and its effect on specific amino acid residues, molecular docking followed by 100 ns molecular dynamics simulations were performed (Fig. 4A). The 100 ns molecular dynamics (MD) simulation results indicated that the RMSD fluctuations of the protein gradually decreased over time and eventually stabilized with minor oscillations, suggesting that the system had reached equilibrium and convergence (Fig. 4B). Simultaneously, a gradual decrease in the radius of gyration (Rg) and solvent accessible surface area (SASA) indicated progressively tighter binding between the protein and MoS_2_ nanosheets (Fig. 4C and S2C). Analysis of root mean square fluctuation (RMSF) revealed that conformational adjustments in flexible regions of the protein facilitated closer association with MoS_2_ (Fig. S2D). Interaction energy analysis demonstrated that both van der Waals and Coulombic interactions contributed favorably to the binding process, with van der Waals interactions playing a more dominant role (Fig. 4D).

In addition, Fig. 4E–F confirmed that both endogenous MST2 protein and purified MST2 recombinant protein could be pulled down by MoS_2_ nanosheets. We added fluorescein isothiocyanate-bovine serum albumin (FITC-BSA) labeled MoS_2_ nanosheets into cells, and found that FITC-labeled MoS_2_ could colocalized with MST2 through immunofluorescence analysis (Fig. 4G). Together, these results demonstrated that MST2 protein might directly bind to MoS_2_ nanosheets. We further determined the binding constants of MoS_2_ and MST2 protein by Isothermal Titration Calorimetry (ITC). As shown in Fig. 4H, when titrating a suspension of MST2 with the solution of MoS_2_ nanosheets, positive peaks were displayed, indicating that MoS_2_ immobilization into MST2 proceeds *via* an endothermic pathway (KD = 5.91e-6±7.50e-9; TΔS = 41 kJ/mol). In addition, the secondary structures of the MST2 protein changed after incubated with MoS_2._ After the incubation, the α-helical structure decreased by 3.66 %, and the β-sheet content of MST2 protein increased by 5.29 % as measured by FTIR (Fig. 4I and Table S2). Together, these data demonstrated that MoS_2_ could adsorb MST2 protein to its surface and change the conformation of MST2 protein.

Alkaline phosphatase (AP) catalyzes the hydrolysis of a wide variety of phosphate group from proteins to decrease the protein phosphorylation levels. To explore how the binding of MST2 and MoS_2_ affect the phosphorylation of MST2, we incubated alkaline phosphatase with cell lysis harvested from control or MoS_2_-treated cells. As shown in Fig. 4J, pretreated with MoS_2_ stabilized the phosphorylation level of MST2 protein when added with a series dose of alkaline phosphatase, indicating that MoS_2_ nanosheets affects the phosphorylation level of MST2 by decreasing its dephosphorylation rate.

Docking analysis identified the distinct binding sites may at residues Q389A, V388A, Q489A, Q488A, F231A, N236A, T235A, N236A, R228A, R390A, F491A, F402A (Fig. 4A). Then we generated both the wild-type (WT) and its mutant (Mut) plasmids with alanine substitutions at binding sites. As shown in Fig. S2E–F, FITC-labeled MoS_2_ colocalized with MST2 was significantly reduced in cells transfected with binding site mutants MST2 plasmid, and mutant MST2 protein could not be pulled down by MoS_2_ nanosheets in cell lysate. These results confirm that the binding of MoS_2_ nanosheets to MST2 protein depends on specific amino acid residues.

### 2.6 MoS_2_ nanosheets inhibit cell proliferation through Hippo pathway

**Fig. 5:**
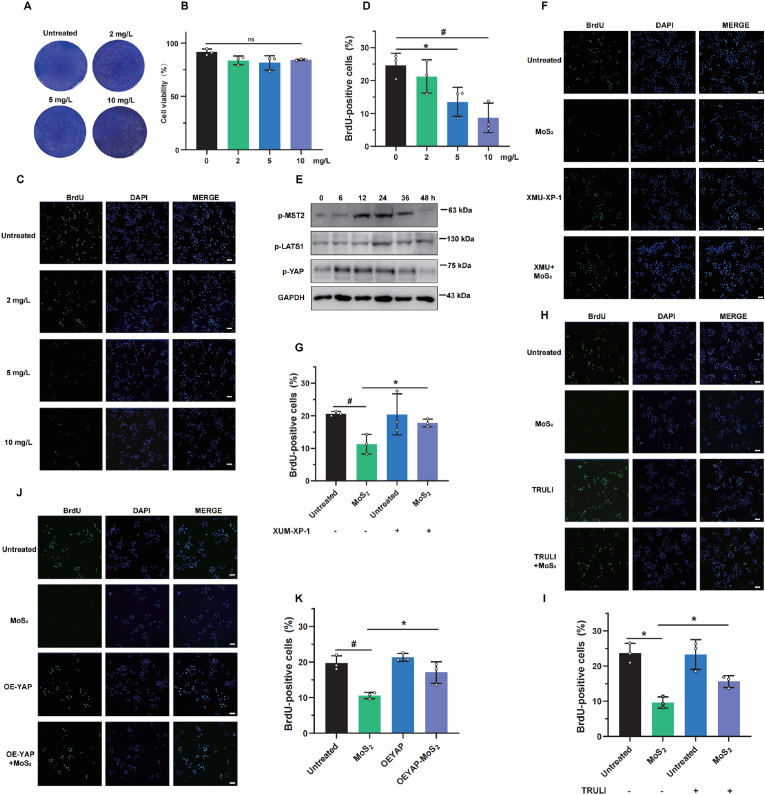
**MoS_2_ nanosheets inhibit cell proliferation through the Hippo pathway.** (A) Crystal violet staining of HepG2 cells treatment with the 2, 5, 10 mg/L the MoS_2_ nanosheets. (B) Trypan blue exclusion of HepG2 cells treatment with the 2, 5, 10 mg/L the MoS_2_ nanosheets (n = 3). (C) Representative fluorescence images of BrdU-positive cells after HepG2 cells treatment with the 2, 5, 10 mg/L the MoS_2_ nanosheets for 24 h. Scale bar = 100 μm. (D) The quantitative analysis of BrdU-positive cells (n = 3). (E) The protein levels of phosphorylated MST2, LATS and YAP in cells treatment with the 5 mg/L the MoS_2_ nanosheets at different time points were assessed by western blotting assay, the experiments were performed 3 times independently. (F–I) Representative fluorescence images and the quantitative analysis of BrdU-positive cells in the control and pretreatment groups (XMU-XP-1 or TRULI pretreated) treated with PBS or MoS_2_ nanosheets for 24 h. Scale bar = 100 μm. (J–K) Representative fluorescence images and the quantitative analysis of BrdU-positive cells in the control and YAP-overexpressing cells treated with PBS or MoS_2_ nanosheets for 24 h. Scale bar = 100 μm. A two-sided Student's t-test was used to determine p-values (∗p < 0.05, #p < 0.01).”. (For interpretation of the references to colour in this figure legend, the reader is referred to the Web version of this article.)

In light of MST2's pivotal role in cell proliferation, we explored whether MST2, influenced by MoS_2_, exerted an influence on hepatocyte proliferation. As shown in Fig. 5A–D, exposure to MoS_2_ at the dosages of 2 and 5 mg/L had no effect on cells survival rate but significantly reduced the number of BrdU-positive cells, indicating that MoS_2_ indeed interferes with cell division and proliferation. Meanwhile, MoS_2_ treatment also induced the activation of Hippo pathway (Fig. 5E), suggesting that Hippo pathway is critical for MoS_2_-induced cell proliferation inhibition. Indeed, both LATS1/2 inhibitor TRULI and XMU-XP-1 increased the number of BrdU-positive cells induced by MoS_2_ (Fig. 5F–I). Notably, a marked increase in BrdU-positive was observed in cells overexpressing YAP- a core effector of the Hippo pathway-following treatment with MoS_2_ nanosheets, further underscoring the essential role of Hippo signaling in MoS_2_-induced hepatotoxicity (Fig. 5J–K). Together, these results indicated that MoS_2_ nanosheets can inhibit cell proliferation through the MST2-dependent Hippo pathway.

### 2.7 MoS_2_ nanosheets inhibit mice liver regeneration through MST2

**Fig. 6:**
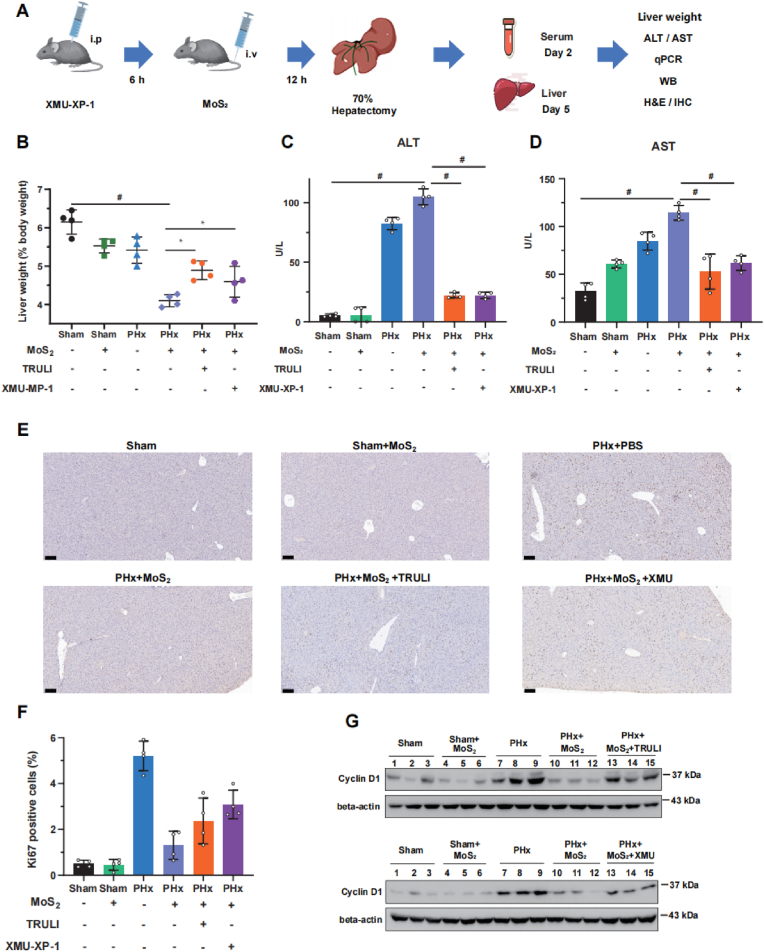
**MoS_2_ nanosheets inhibit mice liver regeneration through MST2.** (A) Control group or drug treatment (XMU-XP-1 or TRULI) group of C57BL/6 mice were exposed to PBS, 1 mg/kg of MoS_2_ nanosheets, and then half of the mice in each group underwent 70 % hepatectomy. After 2 days, the activity of ALT/AST in serum were measured. After 5 days, the liver tissues collected and weighed then qPCR, WB, and IHC staining were performed. (B) Liver to body weight ratios of all group. Sham: Sham surgery group; PHx: Partial hepatectomy group. (C–D) The activity of ALT/AST in serum were measured by ALT/AST assay kit (n = 4). (E) Immunohistochemistry for Ki-67 of all group C57BL/6 mice liver. Scale bar = 100 μm. (F) Positive nuclei of Ki-67 were quantified with ImageJ software. (G) The protein levels of Cyclin D in liver were detected by Western blotting assay; the experiments were performed 3 times independently. A two-sided Student's t-test was used to determine p-values (∗p < 0.05, #p < 0.01).

Hepatocyte proliferation is the main mechanism for liver regeneration. Therefore, we used a classic model of 70 % hepatectomy that the remaining liver lobes restore the original liver mass within one week after liver resection to confirm the inhibition effect of MoS_2_ nanosheets on hepatocyte proliferation *in vivo*. As shown in Fig. 6A–B, treatment of MoS_2_ resulted in a slower recovery of liver weight in mice after surgery, which was rescued by the addition of TRULI or XMU-XP-1. In addition, the increased serum ALT and AST levels in MoS_2_-treated mice were higher than the untread group after two days post-operation, which could be also reversed when MST2 and LATS1/2 activation were inhibited (Fig. 6C–D), indicating that MoS_2_ treatment caused delay and attenuation of regeneration through Hippo pathway. Moreover, Ki-67 expression staining indicated that MoS_2_ nanosheets exposure decreased the regeneration capacity the of the liver, whereas pretreated with TRULI or XMU-XP-1 increased the number of Ki-67-positive hepatocytes in regenerating liver of MoS_2_-treated mice; meanwhile,the expression levels of Cyclin D1, an important cyclin protein involved in regulating cell cycle progression, also exhibited the same tendency as Ki-67 (Fig. 6E–G). Taken together, these results demonstrated that MoS_2_ treatment indeed inhibited the proliferation of hepatocytes in regenerating liver by regulating the activation of MST2.

### 2.8 MoS_2_ nanosheets inhibit zebrafish liver development through MST2

It was reported that Hippo pathway plays a major role in liver development and both loss of MST1 and MST2 results in uncontrolled liver overgrowth. Therefore, we hypothesized that MoS_2_ might also affect liver development. To this end, we utilized a zebrafish line *Tg* *(-1.7apoa2: GFP)* that marks liver organogenesis as a model to evaluate the effect of MoS_2_ on the developmental phases of the zebrafish liver. As depicted in Fig. S3A–C, GFP-positive cells in the liver primordium were clearly observable at 3 days post-fertilization (dpf) and the liver was enlarged with almost 100 % penetrance in the control group; however, exposure of embryos to MoS_2_ significantly reduced the intensity of GFP fluorescence in zebrafish liver, in addition, the recovery phenomenon was observed when added with TRULI or XMU-XP-1. Together, our date indicated that MoS_2_-induced MST2 phosphorylation is indispensable for its inhibitory effect on liver development.

### 2.9 MoS_2_ induces autophagy-dependent cell death *via* MST2-LC3B pathway

**Fig. 7:**
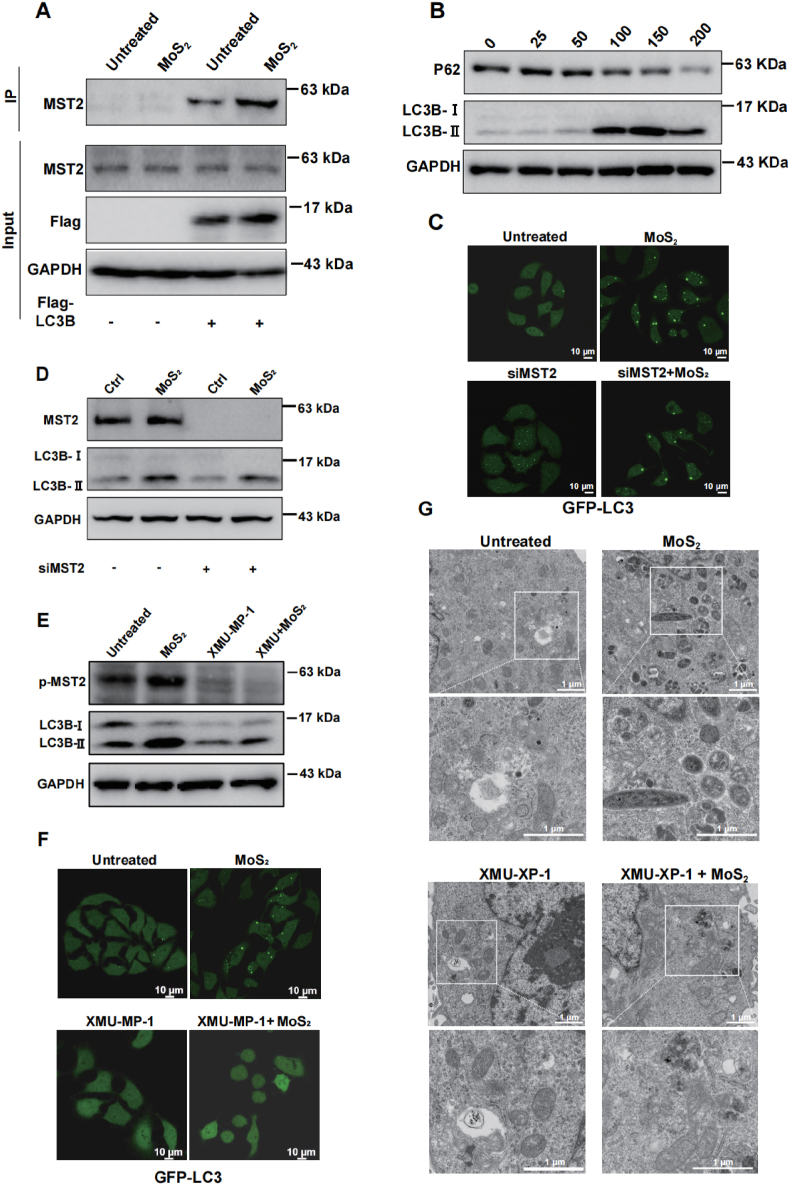
**MoS**_**2**_**induces autophagy-dependent cell death*****via*****MST2-LC3B pathway.** (A) HepG2 cells were transfected with full length Flag-LC3B plasmid, cell lysates were then incubated with anti-FLAG beads overnight at 4 °C, Western blot was then performed to detect the protein level of MST2. (B) Western blot analysis of the protein levels of autophagy related indicators (LC3B and P62) in the HepG2 cells treated with indicated concentration of MoS_2_ nanosheets for 24 h, GAPDH was used as a control, the experiments were performed 3 times independently. (C) Representative images of the accumulation of GFP-LC3 puncta in the control and MST2-depleted cells treated with PBS or 100 mg/L MoS_2_ nanosheets. Scale bar = 10 μm. (D) Western blot analysis of the protein levels of LC3B and MST2 in control and MST2-depleted cells treated with PBS or 100 mg/L MoS_2_ nanosheets, the experiments were performed 3 times independently. (E) Western blot analysis of the protein levels of LC3B and MST2 in control and inhibited phosphorylation MST2 (XMU-XP-1 pretreated) cells treated with PBS or 100 mg/L MoS_2_ nanosheets, the experiments were performed 3 times independently. (F) Representative images of the accumulation of GFP-LC3 puncta in the control and inhibited phosphorylation MST2 (XMU-XP-1 pretreated) treated with PBS or 100 mg/L MoS_2_ nanosheets. Scale bar = 10 μm. (G) Representative electron microscopy images of the cells in control and inhibited phosphorylation MST2 (XMU-XP-1 pretreated) cells treated with PBS or 100 mg/L MoS_2_ nanosheets. Scale bar = 1 μm.

MST2 is one important component of classical Hippo pathway to regulate the nuclear translocation of YAP/TAZ. Our data indeed showed that the phosphorylation levels of large tumor suppressor 1/2 (LATS1/2), a downstream target of MST2, and YAP, the key effector of the Hippo pathway, were increased under MoS_2_ treatment (Fig. 5E and S4A); meanwhile, the nuclear translocation of YAP was also decreased upon MoS_2_ exposure (Fig. S4B), the inhibitors of AMPK or DNA-PK did not change the translocation of YAP, indicating that MoS_2_ induced the activation of Hippo pathway specifically. Both YAP overexpression and attenuated YAP phosphorylation achieved by the TRULI inhibitor exerted a partial yet protective effect against MoS_2_-induced cell death (Fig. S4C–H). However, a considerable number of cells remained unrescued. This indicates that Hippo pathway activation is not the primary cause of cell death induced by MoS_2_. It was also reported that MST2 is also important for autophagy through directly phosphorylating LC3B [11,12], we thus hypothesized that MoS_2_ might affect autophagy through MST2-LC3B pathway. Co-IP assay was performed to test whether MoS_2_ affect the interaction between MST2 and LC3B. As shown in Fig. 7A, their interaction was enhanced upon MoS_2_ treatment, indicating that LC3B is the downstream substrate of MoS_2_/MST2 complex. In addition, the total levels of LC3-II were dramatically induced, whereas p62 levels were significantly reduced when in response to MoS_2_ exposure (Fig. 7B); meanwhile, MoS_2_ treatment led to a robust accumulation of GFP-LC3 puncta (Fig. 7C), indicating that MoS_2_ exposure enhances autophagic flux in cells. Moreover, both downregulation of MST2 expression by siRNA and inhibition of MST2 phosphorylation by XMU-XP-1 significantly reversed MoS_2_-upregulated autophagic flux (Fig. 7C–F). Furthermore, a large amount of autolysosomes were found in cells treated with MoS_2_ nanosheets, and XMU-XP-1 pretreatment markedly reduced the formation of autolysosome significantly (Fig. 7G). The above results demonstrated that MoS_2_ nanosheets induced autophagy through MST2-LC3B pathway.

To further confirm the role of autophagy in MoS_2_-induced cell death effect, autophagy was inhibited by using siRNA ATG5 (participate in the formation of autophagosomes) and inhibitor BFA (Bafilomycin A1, a macrolide antibiotic, thereby hindering the union of autophagosomes with lysosomes within the cell). As shown in Fig. S5A–H, when MoS_2_ treatment-enhanced LC3-II expression and GFP-LC3 puncta formation were inhibited, the death cell effect was also significantly reversed, indicating that MoS_2_-induced autophagy is an important cause of cell death.

**Fig. 8:**
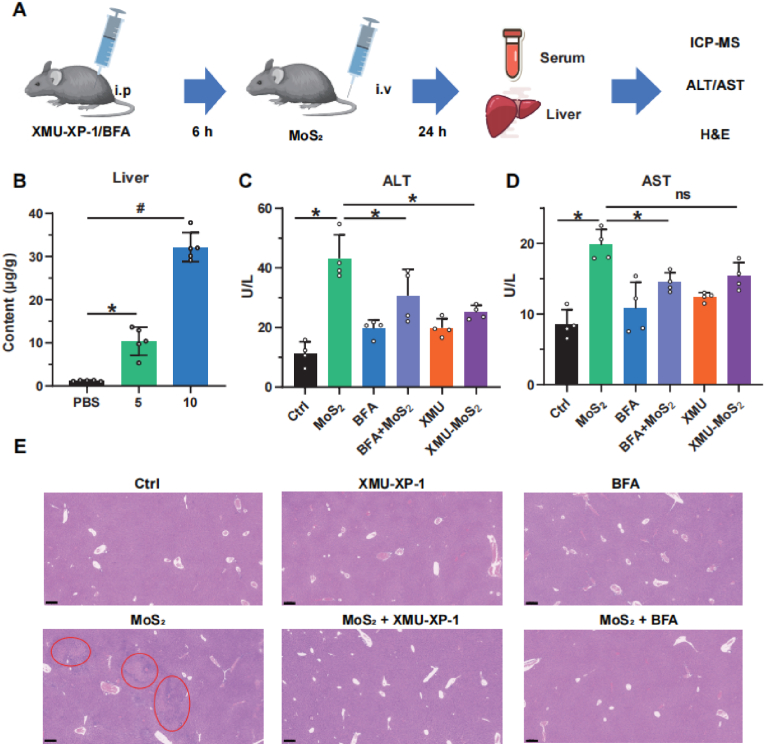
**MoS**_**2**_**nanosheets exposure induces autophagic acute liver injury in mice.** (A) Control group or inhibitors (XMU-XP-1 or BFA) pretreatment group of C57BL/6 mice were exposed to PBS, 5 and 10 mg/kg of MoS_2_ nanosheets or by tail vein injection. After 24 h, the liver tissue and serum were collected. ICP-MS for molybdenum, activity of ALT/AST and H&E staining were performed. (B) The content of molybdenum in liver tissues of the control or MoS_2_ exposure group (n = 4). (C–D) The activity of ALT/AST in serum were measured by ALT/AST assay kit (n = 4). (E) Liver tissues of mice were sliced and then stained with hematoxylin-eosin for histopathologic examination, scale bar = 250 μm. (The red circled area represents the damaged tissue). A two-sided Student's t-test was used to determine p-values (∗p < 0.05, #p < 0.01). (For interpretation of the references to colour in this figure legend, the reader is referred to the Web version of this article.)

*In vivo* experiments were further performed to verify whether MoS_2_ causes acute liver injury through MST2-LC3B pathway. A shown in Fig. 8A, C57BL/6 mice were treated with the nanosheets *via* tail vein injection following intraperitoneal injection of the autophagy inhibitor BFA or the MST1/2 inhibitor XMU-XP-1. The ICP-MS results showed that there was a significant accumulation of molybdenum ions in the liver (Fig. 8B), indicating that a large amount of MoS_2_ could accumulate in and cause potential damage to the liver. In addition, MoS_2_ exposure-induced increase of serum ALT and AST levels, as well as the pathological changes determined by H&E staining, were all reversed when pretreatment with BFA or XMU-XP-1 (Fig. 8C–E), indicating that MoS_2_-induced acute liver injury was also through MST2/LC3B axis.

## 3 Discussion

Due to their simple preparation method, biocompatibility, and excellent NIR light absorption performance, 2D MoS_2_ nanosheets are increasingly applied for biomedical application; however, the potential cytotoxicity and health risk of MoS_2_ has been largely overlooked. After entering the body, the 'nano-bio interactions' can affect the biodistribution and biotransformation of MoS_2_, which determines its biocompatibility.

The large surface area to volume ratio, high surface free energy, ultrathin structure and hydrophobicity enable MoS_2_ to more easily prone to adsorb and affect the biological functions of proteins, which probably leading to dysregulated outcomes such as cell proliferation, metabolism, differentiation, and cell death. The cytotoxicity of MoS_2_ nanosheets is critically influenced by their physicochemical properties, including size, surface charge, and layer number. These physicochemical parameters dictate bio-nano interactions, cellular uptake, biodistribution, and metabolic fate, ultimately determining hepatic damage mechanisms [13]. For example, small size of MoS_2_ can easily enter hepatic sinusoidal endothelial cells through blood circulation and be engulfed by Kupffer cells. Size of 50–200 nm MoS_2_ nanosheets accumulate predominantly in the liver of mice, triggering lipid dysregulation, inflammation, and atherogenesis after long-term exposure [14]. But, micron-sized MoS_2_ may accumulates in lungs, causing more severe pulmonary toxicity [15]. Meanwhile, surface charge determines protein corona composition, influencing hepatic sequestration. Negative surfaces enhance ApoE-mediated hepatic uptake; PEGylation (charge neutralization) mitigates protein adsorption and accumulation [16,17]. And the more exfoliated the MoS_2_ nanosheets, the stronger its cytotoxic influence, which may be due to an increase in surface area and active edge sites [18]. Therefore, it is necessary to clarify the standardized degradation kinetics model of MoS_2_ *in vivo*, and to balance treatment needs and liver safety in drug delivery design.

In this study, we found that MoS_2_ exposure caused damage to liver cells *in vitro* and *in vivo* through directly bound to MST2 protein. This interaction promoted the phosphorylation and activation of MST2, resulting in proliferation inhibition through Hippo pathway and autophagy-dependent cell death by regulating LC3B. Therefore, our data provide a compressive understanding of the hepatotoxicity of MoS_2_ and suggest that targeting MST2 is a promising strategy to alleviate MoS_2_-induced health risk. Surface engineering has proven feasible to suppress nanosheets toxicity. Some biocompatible surface coatings could reduce non-specific accumulation of nanoparticles and regulate their degradability in the liver microenvironment. For example, polyethylene glycol of MoS_2_ reduces liver accumulation and hepatotoxicity by reducing protein adsorption and protein crown formation [16]. Although reducing the exposure dose can reduce the hepatotoxicity of MoS_2_ nanosheets, it has been reported that the proportional nanoparticle uptake by the liver would decrease significantly once the dose surpassed maximum Kupffer cell uptake rates by inhibiting the binding efficiency of surface receptors [19]. Therefore, it is necessary to establish a threshold absorption kinetics model and clarify the effects of exposure dose and time on the degradation and hepatotoxicity of MoS_2_ nanosheets *in vivo*, and to expand their applications in biomedicine. And all strategies must be evaluated with regard to their capacity to maintain the therapeutic or diagnostic efficacy of MoS_2_ nanosheets while mitigating adverse effects associated with MST2-mediated growth suppression and cell death.

It was well documented that MST1/2 kinase activity inhibits YAP function to restrict proliferation in mammalian liver. For example, the deletion of MST1/2 can be induced through the intravenous administration of adenoviral vectors encoding Cre recombinase in adult murine livers, leading to the prompt initiation of hepatocellular proliferation [20]. The use of liposomal encapsulation to inhibit MST1/2 has been shown to decrease MST1/2 mRNA levels, stimulate YAP1 activity, and prompt hepatocytes to re-enter the cell cycle, thereby facilitating liver regeneration in elderly mice post-hepatectomy [21]. In our study, we found that MoS_2_ activated the Hippo pathway *via* the phosphorylation of MST2 protein to inhibit cell proliferation *in vitro*, and suppress mice liver regeneration and zebrafish liver development. Therefore, targeting MST2 may represent a promising strategy to alleviate MoS_2_-induced hepatotoxicity. For instance, concomitant administration of the free radical scavenger chlorophyllin has been shown to reduce clastogenicity, genotoxicity, and mutagenicity caused by titanium dioxide (TiO_2_) nanoparticles in mice [22]. Additionally, co-administration of α-lipoic acid with lead and zinc oxide nanoparticles can counteract the neurotoxicity, immunotoxicity, and reproductive toxicity induced by these nanoparticles [23]. These examples support the potential of combinatorial approaches in nanotoxicology. Thus, the concomitant application of MST2 inhibitors with MoS_2_ nanosheets could offer a viable therapeutic avenue to reduce their hepatotoxic effects.

Autophagy is an effective approach to degrade and clear nanomaterials in cells. Although autophagy is primarily considered to be a cell survival mechanism, excessive autophagy flux induced by nanomaterials might have adverse effects on cells, such as clearing excessive cytoplasmic substances and causing severe morphological changes and autophagic cell death. For example, functionalized single-walled carbon nanotubes induced autophagic cell death in A549 cells by modulating the AKT-TSC2-mTOR signaling axis, and were also implicated in the induction of acute pulmonary damage *in vivo* [24]. Polyamide amine dendrimers induced an increase of autophagy flux in human glioma cells through Akt/mTOR/p70S6K pathway, leading to abnormal accumulation of autophagosomes and neurotoxicity [25]. Human monocytes exposed to CeO_2_ NPs exhibited significant autophagic cell death by activating PI3K pathway [26]. It was reported that key autophagy factors LC3 was a novel substrate of MST1/MST2, and both MST1 and MST2 can phosphorylate LC3 at Thr50 to regulate the fusion of autophagosomes and lysosomes [11]. Our results also showed that MoS_2_ exposure promoted the increase of LC3B-II through MST2, which led to an increases of intracellular autophagy flux and subsequently triggered autophagic cell death *in vitro* and *in vivo*. However, the molecular mechanism of MST2 regulating LC3 phosphorylation upon MoS_2_ exposure still requires further investigation.

## 4 Conclusions

**Fig. 9:**
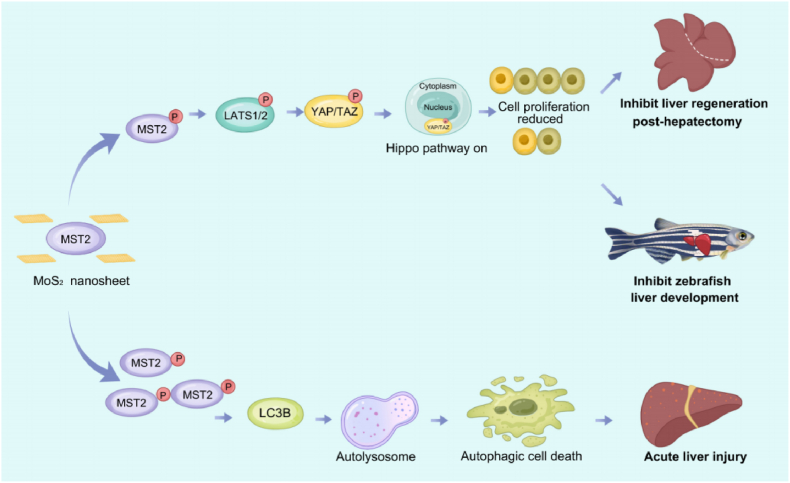
**The proposed model underlying the possible molecular mechanism of MoS**_**2**_**-induced hepatotoxicity.**

This study indicated that MoS_2_ could cause hepatotoxicity *in vitro* and *vivo*, and identified that MST2 protein could directly interact with MoS_2_. Moreover, MoS_2_ inhibited liver cell proliferation, mice liver regeneration and zebrafish liver development by regulating MST2 and Hippo pathway. Meanwhile, we also found that MoS_2_ induced the autophagy-dependent cell death and mice acute liver injury *via* MST2-LC3B pathway (Fig. 9). This is the first report to our knowledge that MoS_2_ can induce nonnegligible liver damage through regulating MST2-meidated pathways, which also provides scientific basis and promising strategy to ensure the safer clinical application of MoS_2_ in the future.

## 5 Experimental methods

### 5.1 Synthesis of MoS_2_ nanosheets

70 mL of deionized water, 40 mg of MoCl_5_ (Aladdin, Shanghai, China) and 57 mg of thiourea (Sigma-Aldrich, Shanghai, China) were added into a conical flask. After 1 min of ultrasonication, the mixture was heated to 200 °C for 24 h. Once cooled to room temperature the mixture was washed three times with deionized water, then dried at 60 °C.

### 5.2 Transmission electron microscopy (TEM)

MoS_2_ nanosheets were dissolved in PBS and sonicated for 30 min. A 200-mesh ultra-thin copper grid (Mirror Technology, Beijing, China) was placed in a 12-well culture plate. Then, 10 μL of the MoS_2_ solution was dropped onto the copper grid, allowed to dry in a 37 °C incubator, and subsequently observed with a transmission electron microscope (TEM; JEOL, Japan).

### 5.3 X-ray photoelectron spectroscopy (XPS)

The MoS_2_ solution was sonicated for 30 min and then several drops were dropped onto the slide of a silicon wafer. The silicon wafer was dried at room temperature. The spectroscopy data were obtained from the X-ray photoelectron spectrometer (Thermo Fisher Scientific, Waltham, USA).

### 5.4 Dynamic light scattering

The size and ζ-potential of the MoS_2_ solution were documented utilizing a laser-based particle size analyzer from Malvern Instruments (Malvern, UK). This equipment facilitated the determination of particle size through dynamic light scattering techniques and the assessment of the ζ-potential.

### 5.5 Atomic force microscopy (AFM)

The thickness of the MoS_2_ nanosheets was determined through AFM measurement (Bruker, Bremen, Germany). Simply, the MoS_2_ powder sample was fixed on the holder using the adhesive tape method. Use the AFM's accompanying data processing software to analyze the obtained image and extract the average height.

### 5.6 Cell culture

HepG2 cells, purchased from the Cell Resource Center of the Institute of Basic Medical Sciences (CAMS, Beijing, China), was cultured in Dulbecco's Modified Eagle Medium (DMEM) enriched with 10 % fetal bovine serum (FBS). The cells were kept in an incubator set at 37 °C with a 5 % CO_2_

### 5.7 Plasmids, RNA interference and lentiviral

The siRNAs for *MST2* and *ATG5* were synthesized by Genepharma Biotechnology (Suzhou, China). The sgRNA for *MST2* was synthesized by Genescript (Nanjing, China). The full length of human Flag-*LC3B* vector was purchased from MiaoLing Plasmid Platform (Wuhan, China). The full length of human His-*MST2* vector was purchased from Weizhen Biosciences (Jinan, China). HepG2 cell line was subjected to transfection with plasmid DNA or siRNA utilizing Lipofectamine 2000 (Life Technologies, Gaithersburg, USA) as the transfection reagent. GFP-LC3 lentiviral was acquired from Hanbio (Shanghai, China).

### 5.8 Reagents and antibodies

Anti-MST2, p-LATS, β-catenin, His-Tag, β-actin and GAPDH were purchased from Proteintech Group (Wuhan, China). Anti-p-MST2 and LC3 were bought from Cell Signaling Technology (Danvers, USA). Anti-Cyclin D and ATG5 were purchased from ZEN-Bioscience (Chengdu, China). XMU-XP-1, Brefeldin A1 and TRULI were purchased from MCE (Shanghai, China). Chlorpromazine, Dynasore, Genistein, methyl-β-cyclodextrin (MβCD), EIPA, Cytochalasin D Dorsomorphin and KU-57788 were purchased from TargetMol (Massachusetts, USA). The second-fluorescence were purchased from Jackson ImmunoResearch (West Grove, USA).

### 5.9 Cell viability assay

A total of 5,000 HepG2 cells were seeded into each well of a 96-well plate, followed by exposure to varying doses of MoS_2_ for durations of 12, 24, and 48 h. After treatment, each well underwent a double rinse with PBS. Then, a mixture of 10 μL of CCK-8 reagent and 90 μL of DMEM was added to each well, and the plates were incubated at 37 °C in darkness for 2 h. The absorbance was subsequently quantified at a wavelength of 450 nm employing a plate reader (Thermo Fisher Scientific, Waltham, USA).

### 5.10 Reactive oxygen species (ROS) production

A quantity of 5,000 cells was placed into each well of a 96-well plate. 5 μM concentration of the oxidative stress indicator H_2_DCFDA (MCE, Shanghai, China) was dispensed into the wells of the 96-well plate, and the plate was kept in the incubator at 37 °C in a light-protected environment for 30 min. Following the introduction of various concentrations of MoS_2_ for a period of 60 min, a plate reader (Thermo Fisher Scientific, Waltham, USA) was employed to measure the absorbance at the wavelengths of 490/514 nm.

### 5.11 Lactate dehydrogenase (LDH)

HepG2 cells were cultured in a 12-well plate. Post a 24 h interaction with MoS_2_, the supernatant from the cell culture was harvested. After spinning down at 2,000 g for a duration of 5 min, a volume of 20 μL from the supernatant was transferred to a 96-well plate. Subsequently, the components of the LDH detection kit (Nanjingjiancheng, NanJing, China) were incrementally added to the plate following the protocol. The absorbance at 450 nm was then recorded to quantify LDH levels, serving as a biomarker for cellular injury.

### 5.12 Western blotting

The detailed steps of western blotting are as described in the previous article [27]. Following a 24 h treatment with MoS_2_, the cells were disrupted using RIPA lysis buffer. After being kept on an ice bath for 30 min, the cell lysates were gathered *via* centrifugation at 12,000 g for 15 min. Equal volumes of protein samples combined with loading buffer were added to each well of the gel. The proteins were then separated by SDS-PAGE and transferred onto a PVDF membrane. After being saturated with 5 % skim milk, the membrane was allowed to interact with the primary antibody and secondary antibodies. Ultimately, the immunoreactive protein bands were visualized employing ECL reagents (Thermo Fisher Scientific, Waltham, USA).

### 5.13 Crystal violet staining

Cells were placed into 12-well plates. Post a 24-h interaction with MoS_2_, cells were fixed with 4 % paraformaldehyde for 15 min. The cells were subjected to staining with a 0.1 % solution of Crystal violet for 30 min, after which they were again rinsed and allowed to dry in air. The stained cells were then photographed to assess the degree of cell adhesion.

### 5.14 Trypan blue exclusion

Cells seeded in 12-well plates were exposed to MoS_2_ for 24 h, then detached with 0.05 % trypsin–EDTA, centrifuged, and resuspended in PBS. An equal volume of 0.4 % trypan blue (Solarbio, Beijing, China) was added; after 3 min, viable and non-viable cells were counted using a cell counter (Countstar, Shanghai, China).

### 5.15 Cellular raman spectroscopy

HepG2 cells were cultured on cell slides for 24 h and then exposed to MoS_2_ solution. After washing with PBS three times, the cell slides were placed on the confocal micro-Raman spectrometer (Horiba, Kyoto, Japan) for imaging. During the Raman imaging and spectrum collection process, the following parameters were used: an excitation wavelength of 532 nm, a power of 10 %, a collection time of 10 s, and a spectral collection range of 300 to 600 cm^−1^.

### 5.16 Electron microscopic observation of cells

For cellular electron microscopy, cells were prepared by fixation, dehydration, infiltration, and embedding. Cut ultrathin sections, stain with heavy metals for contrast, and mount on grids. Examine under an electron microscope (Hitachi, Tokyo, Japan) and capture images for analysis.

### 5.17 Molecular dynamics (MD) simulations

The MST2 crystal structure was downloaded from RCSB-PDB and optimized *via* Discovery Studio. Molecular dynamics (MD) simulations were performed using the Gromacs 2021 software. The protein was described with the amber99sb force field, while the INTERFACE force field was adopted for MoS_2_. The system was solvated in a periodic box with TIP3P water molecules. Sodium and chloride ions were added to neutralize the system. Energy minimization was first conducted to remove unfavorable atomic contacts. Subsequently, a 100 ps NVT equilibration was carried out to raise the temperature to the target value of 298.15 K. This was followed by a 100 ps NPT equilibration to adjust the system density at 1.0 bar. Production simulations were then executed using the leap-frog integrator with a time step of 2 fs for a total of 50 million steps, resulting in a simulation time of 100 ns. Temperature was controlled *via* the V-rescale thermostat at 298.15 K, and pressure was maintained using the Parrinello-Rahman barostat at 1.0 bar. Trajectory analysis was performed using Gromacs tools, molecular visualization was done with PyMOL (http://www.pymol.org/), and images of RMSD, RMSF, radius of gyration (Rg), and interaction energies were generated with Matplotlib in Python.

### 5.18 Isothermal titration calorimetry (ITC)

To determine the binding affinity, a PEAQ-ITC (Malvern, UK) was employed. The calorimeter's reaction cell was filled with the purified MST2 protein (MCE, Shanghai, China) solution maintained at 37 °C, and 100 mM of MoS_2_ was introduced into the cell 19 times with each injection volume being 2 μL. The dissociation constant (*KD*), binding ratios (*n*), molar Gibbs free energy changes (*ΔG*), enthalpy changes (*ΔH*), and entropy changes (*ΔS*) were analyzed using the MicroCal PEAQ-ITC analyzer software.

### 5.19 Fourier transform infrared spectroscopy (FTIR)

1 mg/mL MoS_2_ nanosheet were mixed with 2 mg/mL purified MST2 proteins solution for 1 h at 37 °C. Then, the mixture was transferred to a 300 kDa centrifugal filter unit and concentrated to a final volume of 100 μL by centrifugation at 2,000 *g*. This process was repeated twice to ensure complete removal of unbound MST2 protein [28,29]. Dried in Vacuum dryer for 12 h. Sample powder were mixed with potassium bromide and hydraulically pressed into sheets. FT-IR Spectrometer (Bruker, Bremen, Germany) was used to detected the FTIR spectra with an average 100 scans per sample.

### 5.20 Immunofluorescence

Cells were placed on glass slides within a 12-well plate. Following a 24 h treatment with MoS_2_, the cells were fixed with a 4 % solution of paraformaldehyde. Subsequently, the cells were permeabilized with 0.1 % Triton-X 100. After that, 2 % solution of BSA was applied to block the cells at ambient temperature for 60 min. The primary antibody was added to the slides, and incubated. The cells were further incubated with a fluorescently labeled secondary antibody at 37 °C. After another round of PBS washing, the cell nuclei were counterstained with DAPI for 5 min. The slides were examined and imaged using EVOS M7000 3D Digital Confocal Imaging System (Thermo Fisher Scientific, Waltham, USA).

### 5.21 Immunoprecipitation

5 × 10^7 cells were prepared to extract 20 μg of protein. Then 10 μL of Flag magnetic beads (MCE, Shanghai, China) was added, followed by vortexing at 4 °C for 12 h. A magnetic tube rack was used to separate the magnetic beads, and they were washed with lysis buffer. Then, 50 μL of elution buffer was added, followed by vortexing at 4 °C for 1 h. Finally, 5 × loading buffer was added and the mixture was boiled for 5 min. The co-precipitation results were detected by Western blot.

### 5.22 Mouse treatment

With the oversight of the Research Ethics Committee at the Research Center for Eco-Environmental Sciences, Chinese Academy of Sciences (a proof of approval is available upon request), male C57BL/6 mice aged between six to eight weeks received an intraperitoneal injection of 1 mg/kg body weight of either DMSO, XMU-XP-1, or BFA, with a volume of approximately 100 μL. Six hours post-injection, the animals were given a tail vein injection of 0.5 mg/kg MoS_2_ or PBS. Subsequently, the mice were euthanized, and liver samples were harvested, sectioned, and processed for Hematoxylin and Eosin (H&E) staining and Inductively Coupled Plasma Mass Spectrometry (ICP-MS) analysis. Additionally, serum was extracted for the assessment of alanine aminotransferase (ALT) and aspartate aminotransferase (AST) levels.

### 5.23 H&E staining

Mouse hepatic tissues were preserved in a solution of paraformaldehyde for a duration of 24 h, which was succeeded by a sequence of processes including dehydration, demineralization, and embedding. Subsequently, the tissues were sectioned and stained. The resulting sections were examined and documented using an optical microscope.

### 5.24 ICP-MS

Mouse liver and kidney tissues should be weighed and cut into equal amounts within a 15 mL centrifuge tube. 1 mL of aqua regia was added, and the mixture is then reacted overnight on a constant temperature digestion instrument. Subsequently, 20 μL of the digestion liquid should be mixed with deionized water. The molybdenum standard and the molybdenum content in the sample were determined using an ICP-MS spectrometer (Agilent 8800, Agilent, Santa Clara, USA).

### 5.25 ALT and AST activity detection

The mouse serum was used to detect the level of ALT and AST. In brief, 5 μL of the mouse serum to be tested in the well. Then all reagents of ALT or AST assay kit (Nanjingjiancheng, NanJing, China) were sequentially added to the plate according to the protocol. The absorbance at 510 nm was measured to analyze the content of ALT and AST as an indicator of liver damage.

### 5.26 Cell proliferation detection

The cells were placed in a 12-well plate and allowed to grow overnight. After a 24 h treatment with MoS_2_, the cells were cultured with a 10 μM BrdU solution for 6 h, followed by rinsing with PBS and fixation using a 4 % paraformaldehyde solution. Next, 0.1 % solution of Triton-X 100 was used for cell permeabilization. Then the DNA was subsequently denatured with DNase I to expose the BrdU epitope. Subsequently, 2 % solution of BSA was applied to block the cells at ambient temperature for 60 min. The anti-BrdU antibody was added to cells, and incubated. The cells were further incubated with a fluorescently labeled secondary antibody at 37 °C. After another round of PBS washing, the cell nuclei were counterstained with DAPI for 5 min. The slides were examined and imaged using a confocal microscope.

### 5.27 Partial hepatectomy (PHx) in mice

With the oversight of the Research Ethics Committee at the Research Center for Eco-Environmental Sciences, Chinese Academy of Sciences, C57BL/6 mice, aged 6–8 weeks, were exposed to DMSO, XMU-XP-1 or TRULI, then exposed to MoS_2_. The mice were anesthetized, and then the abdominal area of each mouse was shaved and disinfected. A small incision was made along the midline of the mouse's abdomen to expose the liver, and the abdomen was gently pressed to exteriorize approximately 70 % of the liver. The corresponding hepatic ligament, including blood vessels and bile duct, was ligated using surgical sutures, and the predetermined lobe of the liver was excised outside the ligation line. After the wound was closed with sutures, the mice were placed on a heating pad to maintain body temperature and were provided with proper postoperative analgesia and care. To evaluate the surgical effect and liver regeneration status, the mice were euthanized, and serum and liver tissues were collected for further analysis on days 2 and 4 post-surgery.

### 5.28 Immunohistochemistry of liver tissue

Hepatic specimens of mice were preserved in a 10 % neutral-buffered formaldehyde solution, subsequently processed and encased in paraffin blocks. Tissue sections were deparaffinized, rehydrated, and antigen retrieval was done with citrate buffer. Endogenous peroxidase was inhibited with 3 % H_2_O_2_, and non-specific binding was minimized with 5 % serum. Primary antibodies were applied, followed by HRP-conjugated secondary antibodies and DAB visualization. Slides were hematoxylin-stained, dehydrated, and cleared. Protein expression was evaluated under a light microscope.

### 5.29 Zebrafish husbandry and treatment

The care and handling of zebrafish were conducted under the ethical guidelines provided by the Ethical Review Board of the Research Center for Eco-Environmental Sciences, Chinese Academy of Sciences. A transgenic zebrafish *Tg* (*−1.7apoa2: GFP*) line was bought from China Zebrafish Resource Center (Beijing, China). The *ihb1*75Tg allele is a transgenic zebrafish line *Tg* (*−1.7apoa2: GFP*) with green fluorescent protein driven by the Apo promoter. The GFP-positive cells could be observed in the triangle liver primordium on the left of embryos. Three hundred fertilized eggs of zebrafish were randomly divided into six groups: control group, MoS_2_ (1 mg/L) exposure group, XMU-XP-1 (1 μM) treated group, the group co-treated with XMU-XP-1 and MoS_2_, TRULI (1 μM) treated group, and the group co-treated with TRULI and MoS_2_. The development status of the embryos was checked daily, and dead embryos and those with developmental abnormalities were selected. Liver development in zebrafish was observed using a stereo fluorescence microscope at 3-, 5- and 7-days post-fertilization (dpf). And the zebrafish at 7dpf were collected for IHC staining.

### 5.30 Statistical analysis

All analyses are represented by the mean ± standard deviation. Each experiment was repeated at least three times. For comparison between two groups, t-tests were used. For multiple groups, one-way ANOVA was used. All statistical analyses were performed using GraphPad Prism 8.0 software.

## CRediT authorship contribution statement

**Zijuan Qi:** Writing – original draft, Visualization, Methodology. **Yuanliang Yan:** Software, Resources, Data curation. **Zhijie Xu:** Validation, Supervision, Formal analysis. **Wei Chong:** Data curation, Conceptualization. **Yuchen Qiu:** Methodology, Investigation. **Xiaofeng Huang:** Software, Resources. **Jiajun Jing:** Visualization, Software, Resources. **Huancai Fan:** Visualization, Software, Methodology. **Qiuju Liang:** Validation, Resources. **Sijin Liu:** Supervision, Conceptualization. **Li Yan:** Supervision, Software, Resources. **Leping Li:** Project administration, Conceptualization. **Ming Gao:** Writing – review & editing, Supervision.

## Funding declaration

This research was supported by the National Natural Science Foundation of China (Grant No. 22222611, 22076212) and the Strategic Priority Research Program of the 10.13039/501100002367Chinese Academy of Sciences (Grant XDB0750300).

## Declaration of competing interest

The authors declare that they have no known competing financial interests or personal relationships that could have appeared to influence the work reported in this paper.

## Data Availability

Data will be made available on request.
