# Supplementary material for: Molybdenum disulfide induces growth inhibition and autophagy-dependent hepatocyte cell death through directly binding and regulating the activity of MST2
Source: Mater Today Bio. 2025 Oct 8;35:102394. doi: 10.1016/j.mtbio.2025.102394 (PMC12547203; doi:10.1016/j.mtbio.2025.102394)
Supplement: Multimedia component 1 [file mmc1.docx]

**Molybdenum disulfide induces** **growth inhibition and autophagy-dependent hepatocyte cell death through directly binding and regulating the activity of MST2**

*Zijuan Qi^1,2,7^,* *Yuanliang Yan^4,7^, Zhijie Xu^5^, Wei Chong^1^, Yuchen Qiu^6^, Xiaofeng Huang**^2,3^, Jiajun Jing^2,3^, Huancai Fan^2,3^, Qiuju Liang^2,3^, Sijin Liu^2,3^, Li Yan^2,3^*,* *Leping Li^1^*, Ming Gao^1,2,3^****

1. Department of Gastrointestinal Surgery, Shandong Provincial Hospital Affiliated to Shandong First Medical University, Jinan, 250021, Shandong, China;

2. State Key Laboratory of Environmental Chemistry and Ecotoxicology, Research Center for Eco-Environmental Sciences, Chinese Academy of Sciences, Beijing, 100085, China;

3. University of Chinese Academy of Sciences, Beijing, 100049, China;

4. Department of Pharmacy, Xiangya Hospital, Central South University, Changsha, 410008, Hunan, China;

5. Department of Pathology, Xiangya Hospital, Central South University, Changsha ,410008, Hunan, China;

6. College of Environmental and Resource Sciences, Fujian Normal University, Fuzhou, 350117, Fujian, China;

7. These authors contributed equally: Zijuan Qi, Yuanliang Yan

Authors for correspondence: YL (liyan@rcees.ac.cn), LL (lileping@medmail.com.cn) and MG ([minggao@rcees.ac.cn](mailto:minggao@rcees.ac.cn)).

**Appendix A Supplementary data**


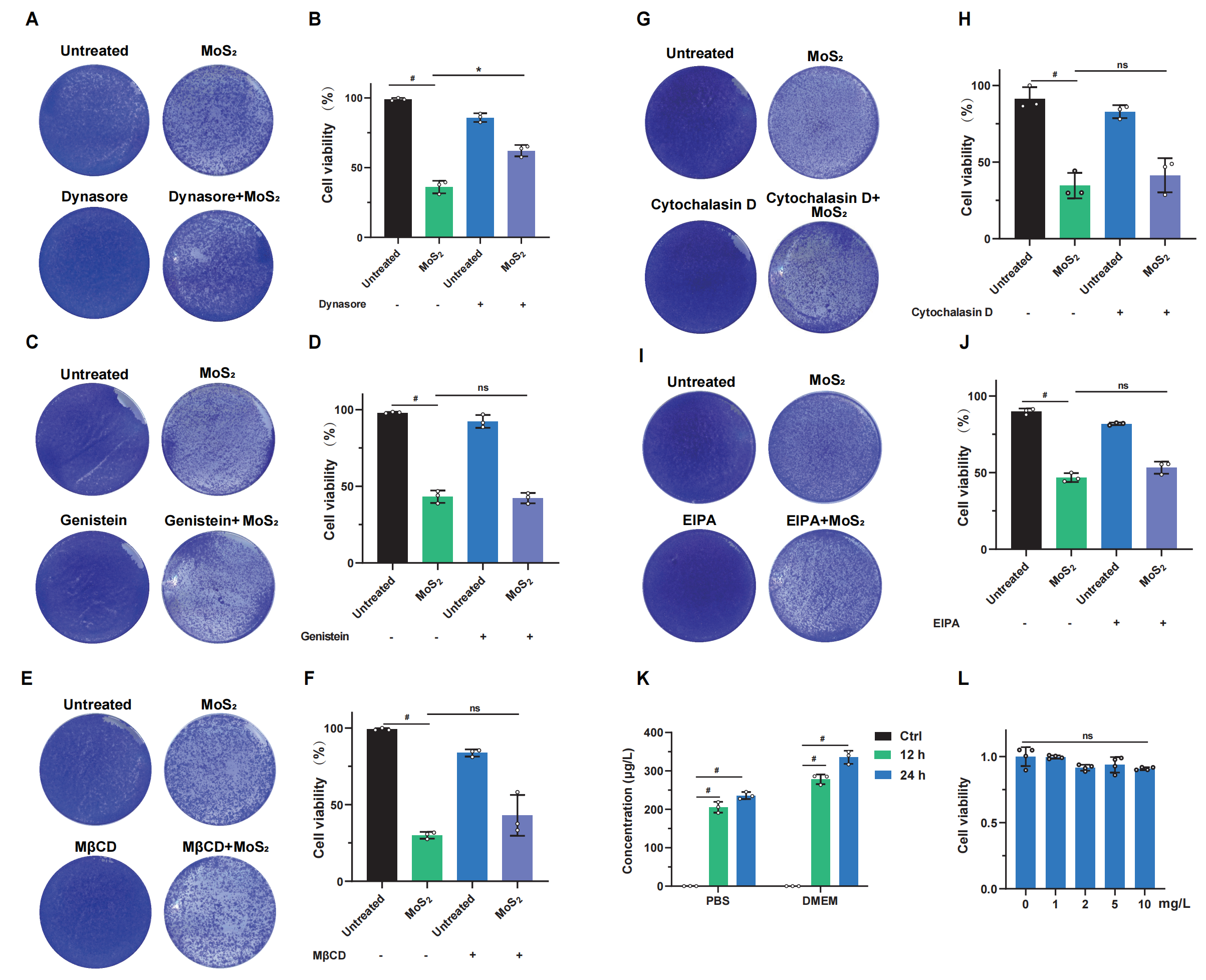


**Fig. S1.** (A) Crystal violet staining of the HepG2 cells treatment with the MoS_2_ nanosheets and clathrin-mediated endocytosis inhibitor (Dynasore). (B) Trypan blue exclusion of the HepG2 cells treatment with the MoS_2_ nanosheets and Dynasore (n = 3). (C) Crystal violet staining of the HepG2 cells treatment with the MoS_2_ nanosheets and Genistein (a caveolin-mediated endocytosis inhibitor). (D) Trypan blue exclusion of the HepG2 cells treatment with the with the MoS_2_ nanosheets and Genistein (n = 3). (E) Crystal violet staining of the HepG2 cells treatment with the MoS_2_ nanosheets and methyl-β-cyclodextrin (MβCD, a lipid raft-mediated endocytosis inhibitor). (F) Trypan blue exclusion of the HepG2 cells treatment with the with the MoS_2_ nanosheets and methyl-β-cyclodextrin (n = 3). (G) Crystal violet staining of the HepG2 cells treatment with the MoS_2_ nanosheets and Cytochalasin D (an inhibitor of F-actin polymerization). (H) Trypan blue exclusion of the HepG2 cells treatment with the with the MoS_2_ nanosheets and Cytochalasin D (n = 3). (I) Crystal violet staining of the HepG2 cells treatment with the MoS_2_ nanosheets and EIPA (a macropinocytosis inhibitor). (J) Trypan blue exclusion of the HepG2 cells treatment with the with the MoS_2_ nanosheets and EIPA (n = 3). (K) The Mo^4+^ concentration in the PBS and DMEM incubated with 200 m/L MoS_2_ solutions for 12 and 24 h were measured by ICP-MS (n = 3). (L) Cell viability of HepG2 cells after treatment with the indicated concentration of molybdenum ions for 24 h were measured by CCK-8 assay (n = 4). A two-sided Student’s t-test was used to determine p-values (* p < 0.05, # p < 0.01).


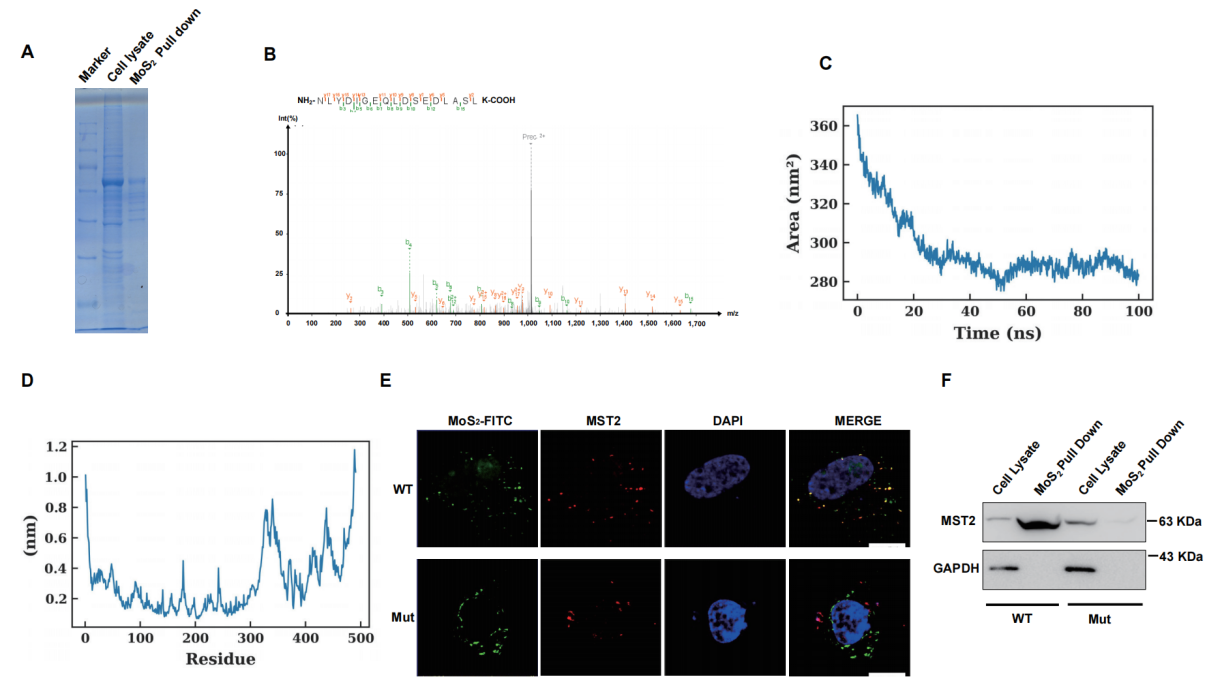


**Fig S2.** (A) Coomassie blue staining of the cell lysis and the cell lysis incubated with MoS_2_ nanosheets. (B) The tandem Mass Spectrum of the peptide of MST2 protein attached to MoS_2_ nanosheets in cell lysate. (C-D) Profiles of solvent accessible surface area (SASA; C) and root mean square fluctuation (RMSF; D) for MST2-MoS_2_ during a 100-ns MD simulation. (E) HepG2 cells were transfected with wild type and binding site mutants MST2 plasmid (Q389A, V388A, Q489A, Q488A, F231A, N236A, T235A, N236A, R228A, R390A, F491A, F402A), then cells were added with 100 mg/L FITC-MoS_2_, and the representative images of MST2 (red) and FITC-MoS_2_ (green) were obtained by immunofluorescence analysis. Scale bar = 10 μm. (F) Lysates from HepG2 cells transfected with wild-type and binding site mutant MST2 plasmids were separately co-incubated with 100 mg/L MoS₂ nanosheets. Proteins bound to the MoS₂ nanosheets were then isolated through washing and membrane ultrafiltration. The bound proteins were detected by Western blot analysis. All experiments were performed independently in triplicate.


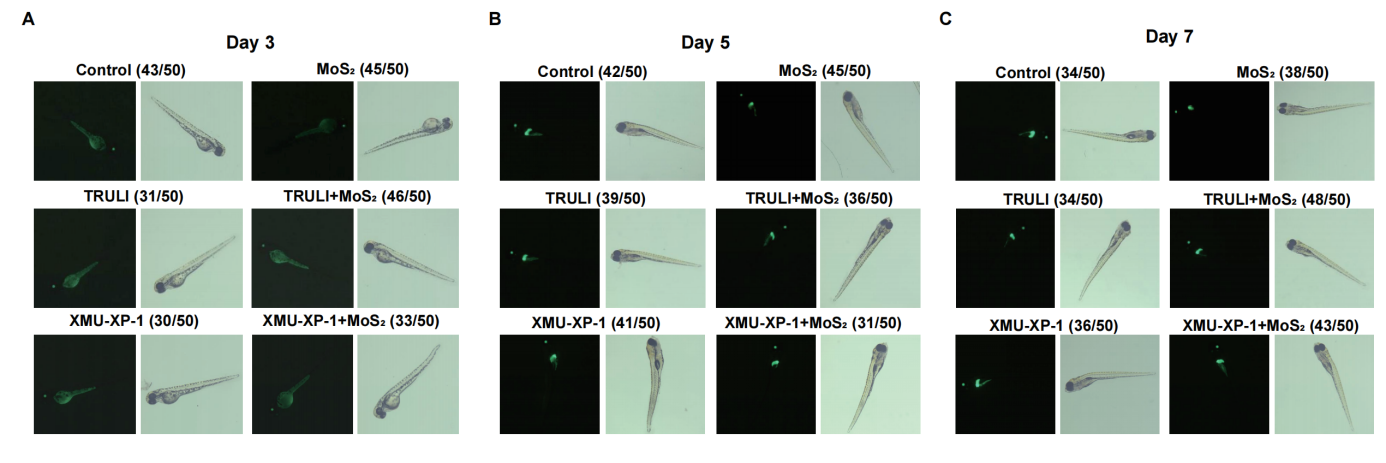


**Figure S3.** (A-C) Control group or drug treatment (XMU-XP-1 or TRULI) group of the zebrafish line Tg (-1.7apoa2: GFP) were exposed to PBS, 1 mg/L of MoS_2_ nanosheets in water. At 3(A)-, 5 (B)- and 7 (C)-days post-fertilization (dpf), the zebrafish of six groups were observed by a stereomicroscope.


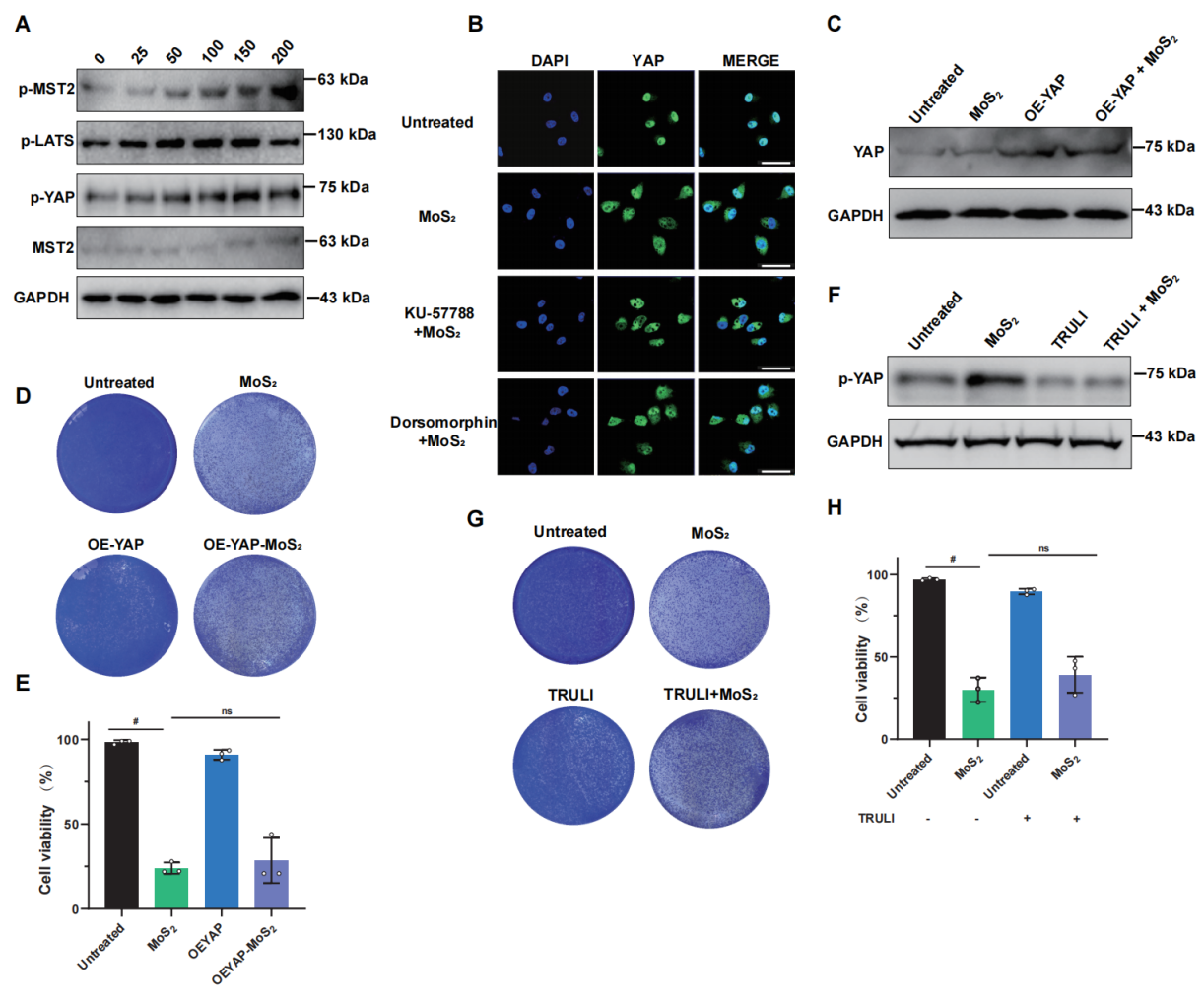


**Fig S4.** (A) The protein levels of phosphorylated MST2, LATS, YAP and MST2 in cells treated with indicated concentration of MoS_2_ nanosheets for 24 h were assessed by Western blotting assay, the experiments were performed 3 times independently. (B) Representative fluorescence images of the YAP and DAPI in the control and pretreatment groups (KU-57788 or Dorsomorphin pretreated) treated with PBS or MoS_2_ nanosheets for 24h. Scale bar = 100  µm. (C) Control or YAP overexpressing group were treated with PBS or 100 mg/L MoS_2_ nanosheets, the protein level of MST2 was then measured by Western blotting assay, the experiments were performed 3 times independently. (D-E) Control or YAP overexpressing group were treated with PBS or 100 mg/L MoS_2_ nanosheets, the living cells were observed by crystal violet staining and trypan blue exclusion (n=3). (F) Control or TRULI pretreatment group were treated with PBS or 100 mg/L MoS_2_ nanosheets, the protein level of p-YAP was then measured by Western blotting assay, the experiments were performed 3 times independently. (G-H) Control or TRULI pretreatment group were treated with PBS or 100 mg/L MoS_2_ nanosheets, the living cells were observed by crystal violet staining and trypan blue exclusion. A two-sided Student’s t-test was used to determine p-values (* p < 0.05, # p < 0.01).


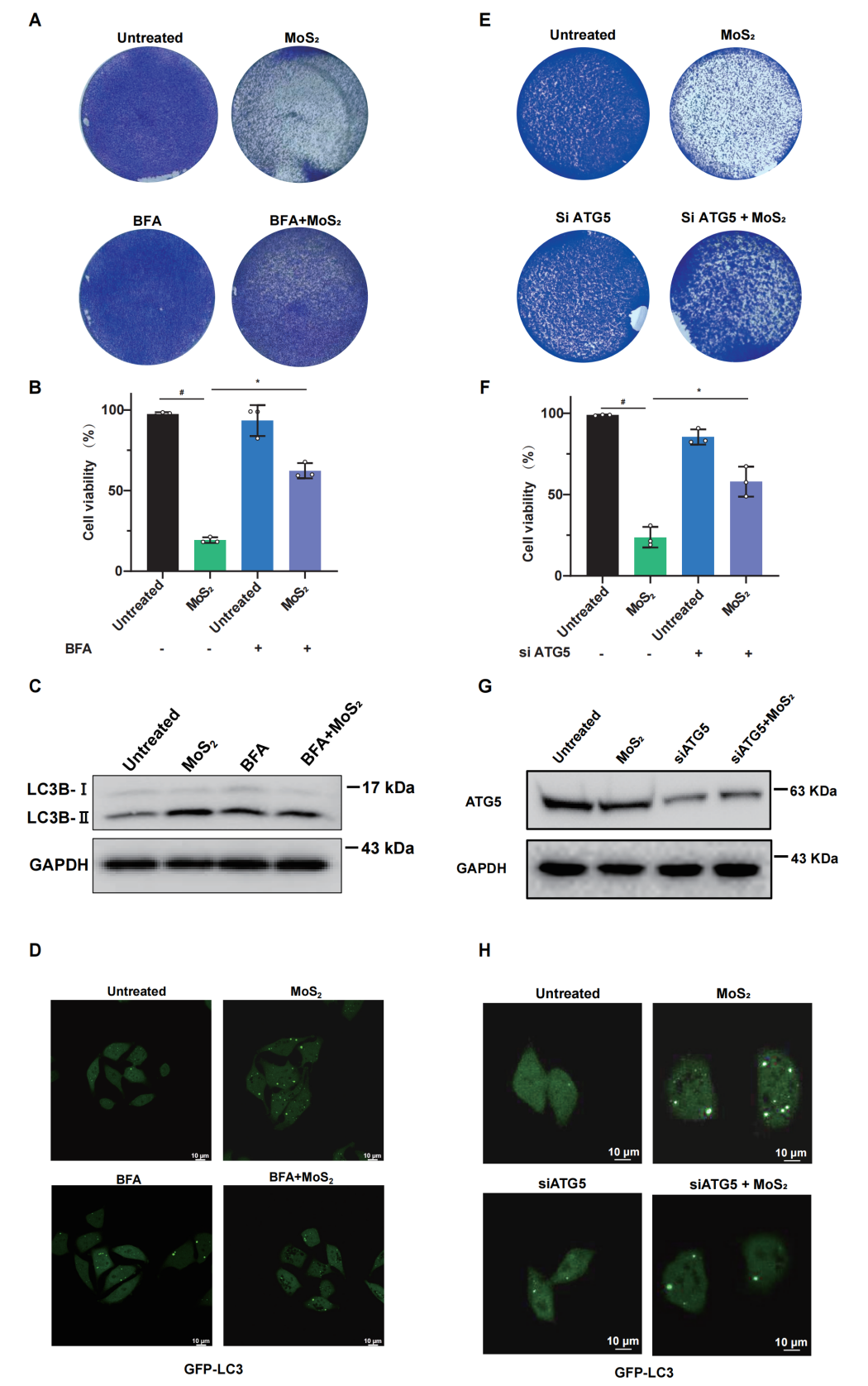


**Fig S5.** (A-D) Control or BFA pretreatment group were treated with PBS or MoS_2_ nanosheets, the living cells were observed by crystal violet staining (A) and trypan blue exclusion (B), the protein level of LC3 were measured by Western blotting assay, the experiments were performed 3 times independently (C). (D) The accumulation of GFP-LC3 puncta in the HepG2 cells were observed by fluorescence microscope. Scale bar = 10  µm. (E-H) Control or ATG5-depleted cells by siRNA group were treated with PBS or MoS_2_ nanosheets, the living cells were observed by crystal violet staining (E) and trypan blue exclusion (F), the protein level of ATG5 were measured by Western blotting assay, the immunoblotting experiments were performed 3 times independently(G), and the accumulation of GFP-LC3 puncta in the ATG5-depleted cells were observed by fluorescence microscope (H), scale bar = 10  µm. A two-sided Student’s t-test was used to determine p-values (* p < 0.05, # p < 0.01).

**Table S1. The result of the protein mass spectrometry on the proteins attached to MoS_2_ nanosheets.**


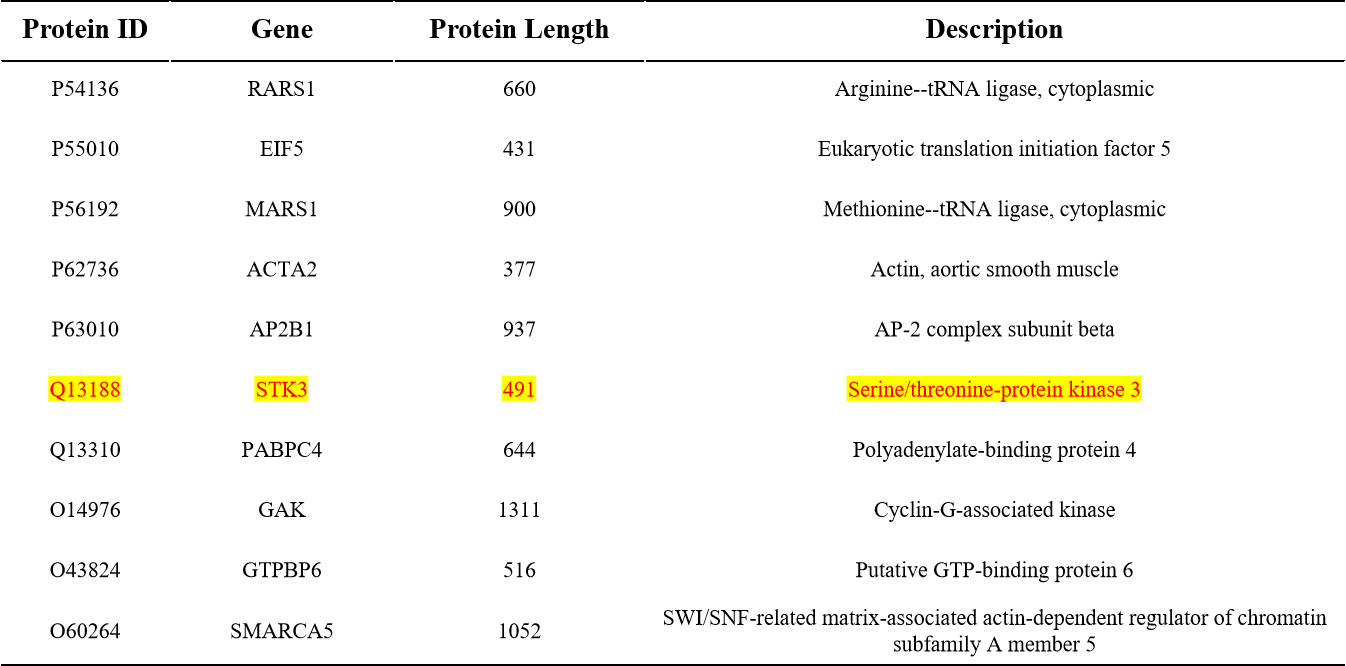


**Table S2. Secondary structure of proteins before and after interacting with MoS_2_ measured by FTIR**

|  | **MST2, %** | **MST2-MoS_2_, %** | **Change, %** |
| --- | --- | --- | --- |
| *α*-helix | 24.87244 | 21.20384 | -3.6686 |
| *β*-sheet | 23.49854 | 28.78882 | 5.29028 |
| *β*-turn | 14.84154 | 10.77424 | -4.0673 |
| Random | 36.78748 | 39.2331 | 2.44562 |
